# Molecular Adaptations and Sex‐Biased Regulatory Pathways in Multi‐Year Diapause of the Cabbage Beetle *Colaphellus bowringi*: A Duration‐Resolved Transcriptomic Analysis

**DOI:** 10.1002/ece3.74603

**Published:** 2026-10-28

**Authors:** Lili Huang, Yan Ni, Xin Ye, Xiaona Liu, Fangsen Xue, Haimin He

**Affiliations:** ^1^ School of Ecology and Environment Yuzhang Normal College Nanchang China; ^2^ Institute of Entomology Jiangxi Agricultural University Nanchang China

**Keywords:** *Colaphellus bowringi*, molecular adaptation, prolonged diapause, sex‐specific regulation, transcriptomics

## Abstract

The cabbage beetle *Colaphellus bowringi*, a key pest of cruciferous crops, exhibits a multiyear dormancy strategy characterized by pronounced interindividual variation in diapause duration: individuals entering summer or winter diapause—though initiated synchronously within each seasonal cohort—may remain in diapause for periods ranging from several months to as long as 5 years. To elucidate the molecular underpinnings of this extended dormancy and its sex‐specific regulatory patterns, we performed a duration‐resolved, sex‐stratified transcriptomic analysis. Adult males and females were sampled after 0.5, 1, and 2 years of diapause durations and subjected to high‐throughput RNA sequencing. *De novo* assembly yielded 66,379 unigenes, of which 16,132 (24.3%) were removed as non‐arthropod contamination, leaving 50,247 unigenes that were used for all downstream annotation and expression analyses. Comparative transcriptomic profiling revealed a conserved adaptive strategy during prolonged diapause: patterns consistent with coordinated suppression of energy‐intensive metabolic processes coupled with increased transcript abundance of genes in cellular protection pathways—including antioxidant defense, xenobiotic detoxification, and protein homeostasis mechanisms. Sex‐biased transcriptional responses were observed: females showed comparable or higher numbers of differentially expressed genes in matched duration comparisons, with sustained transcript abundance of genes associated with reproductive processes and somatic maintenance, whereas males displayed greater duration‐dependent transcriptional remodeling, particularly in signaling cascades and physiological homeostasis networks. KEGG pathway enrichment analysis identified sustained activation of glutathione metabolism, autophagy, and lysosomal degradation pathways throughout diapause; notably, extended diapause (2 years) was specifically associated with significant enrichment of immune response and lipid metabolism pathways. Duration‐dependent dynamics in gene expression underscore that diapause is not a static quiescent state but a highly regulated, multi‐stage process involving duration‐specific transcriptional reconfiguration. This study establishes the first duration‐resolved, sex‐resolved transcriptomic resource for *C. bowringi* and advances mechanistic understanding of long‐term insect diapause—thereby informing the development of targeted, environmentally sustainable pest management strategies.

## Graphical abstract

This study presents the first multi‐duration, sex‐stratified transcriptomic analysis of prolonged diapause in *Colaphellus bowringi*, revealing that extended dormancy is maintained by coordinated metabolic suppression and sustained activation of cytoprotective pathways (e.g., glutathione metabolism, autophagy). Females exhibit greater transcriptional stability with preferential preservation of reproductive functions, whereas males display pronounced plasticity in signaling and metabolic pathways. These findings establish diapause as a dynamic, sex‐divergent regulatory program and provide a key molecular resource for understanding long‐term dormancy in insects.

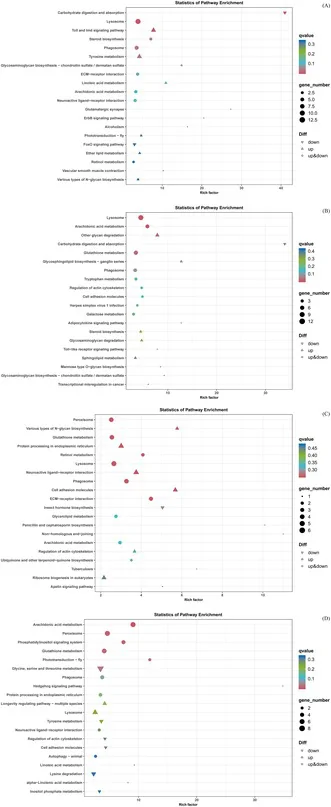

## 1 Introduction

Diapause is a genetically regulated developmental arrest that enables insects to withstand recurrent adverse environmental conditions, including extreme temperatures, drought, and resource limitation (Denlinger 2002; Koštál 2006). Contrary to passive quiescence, diapause entails coordinated physiological and molecular reprogramming, manifesting in metabolic suppression, elevated stress resistance, and developmental suspension (MacRae 2010; Hand et al. 2016). As a critical phenological adaptation, diapause ensures temporal alignment of life cycles with ecologically favorable periods and significantly influences insect population regulation and the emergence of pest outbreaks.

Although typical diapause spans a single unfavorable season, certain insect species undergo prolonged (multi‐year) diapause—a life‐history strategy that staggers adult emergence across multiple years. Danks (1987) documented prolonged diapause in approximately 150 insect species, with durations typically ranging from 2 to 4 years. For instance, in the chestnut weevil *Curculio elephas*, adult emergence is temporally dispersed across three to four consecutive years (Menu 1993). Similarly, in some populations of the Colorado potato beetle ( *Leptinotarsa decemlineata* ), approximately 70% of individuals undergoing prolonged diapause emerge following two overwintering periods (Tauber and Tauber 2002). In the seed‐predatory weevil *Exechesops leucopis*, larval diapause duration exhibits interindividual variation, spanning 1–4 years (Matsuo 2006).

This temporal spreading of emergence functions as a bet‐hedging mechanism, enhancing population resilience to environmental stochasticity and catastrophic disturbances (Hanski 1988; Soula and Menu 2003). Previous studies have indicated that prolonged diapause depletes essential energy reserves—including glycogen and lipids—in various insect species. For instance, in the sawfly *Cephalcia chuxiongica*, both lipid and glycogen stores exhibit a progressive decline throughout extended diapause, thereby constraining the energetic capacity required for post‐diapause stress responses (Xu et al. 2021). In pest species, such extended dormancy poses significant challenges for monitoring and management, as it leads to overlapping generations and persistent, soil‐resident populations (Danks 1987; Šlachta et al. 2002). Consequently, elucidating the molecular and physiological mechanisms that maintain cellular integrity and metabolic quiescence during multi‐year dormancy is essential—not only for advancing fundamental understanding in evolutionary ecology but also for developing effective, evidence‐based pest control strategies.

The cabbage beetle, *Colaphellus bowringi*, is a major agricultural pest of cruciferous crops in Xiu‐Shui County (29°1′N, 114°4′E), Jiangxi Province. This species undergoes both summer and winter diapause in the soil as adults. Its photoperiodic regulation of diapause is strongly temperature‐dependent. At temperatures ≤ 20°C, all adults enter diapause irrespective of photoperiod. Elevated temperatures markedly attenuate the diapause‐inducing effect of long‐day conditions. Conversely, the diapause‐averting effect of short‐day conditions is only observed at temperatures above 20°C. Field monitoring reveals two distinct annual population peaks: one in spring (March–April) and another in autumn (late August to early December) (Xue et al. 2002). Notably, individuals entering diapause in different seasons—spring (April) for summer diapause and autumn (November) for winter diapause—do so under the same low‐temperature conditions (≤ 20°C), yet exhibit substantial interindividual variation in diapause duration, ranging from several months to up to 5 years (Tang et al. 2022). This intraspecific variation in diapause duration is thought to represent a bet‐hedging strategy, enhancing population resilience in unpredictable environments (Soula and Menu 2003). Moreover, females of *C. bowringi* exhibit significantly greater body size than males—a pattern consistent with the well‐documented female‐biased sexual dimorphism prevalent across insect taxa (Tang et al. 2017). This sexual dimorphism, together with the species' extended diapause plasticity, provides a compelling system for investigating sex‐specific life‐history strategies during prolonged dormancy. Consequently, understanding the mechanisms sustaining such extended dormancy has significant implications for both evolutionary ecology and the development of targeted pest management strategies.

Historically, research on insect diapause has centered on environmental cues—such as photoperiod and temperature—and endocrine regulatory mechanisms, particularly the roles of juvenile hormone and ecdysteroids (Denlinger 2002; Izadi 2025). More recently, advances in multi‐omics technologies have enabled systematic characterization of the transcriptional and metabolic reprogramming that underpins diapause across diverse insect species (Cedden et al. 2025). These studies have identified evolutionarily conserved physiological strategies, including metabolic depression, enhanced oxidative stress resistance, and immune system modulation (Poelchau et al. 2013; Ragland et al. 2019; Xiao et al. 2025). For example, comparative transcriptomic analyses in the Asian tiger mosquito, *Aedes albopictus* , have revealed distinct gene expression signatures distinguishing diapause from quiescence—particularly implicating active regulation of lipid metabolism and stress‐response pathways (Poelchau et al. 2013). Nevertheless, the majority of existing work addresses discrete phases of diapause—namely initiation or termination—or examines single‐season dormancy, and even studies sampling more than one time point generally compare a small number of endpoints rather than following a staged or continuous series (Koštál 2006). What remains missing is a comparative analysis that resolves molecular dynamics across multiple successive years of diapause and explicitly tests for sex‐specific differences in these trajectories.

To bridge these critical knowledge gaps, this study reports the first duration‐resolved, sex‐stratified transcriptomic investigation of prolonged adult diapause in the non‐model beetle *C. bowringi* . We systematically compared genome‐wide gene expression profiles between males and females subjected to diapause durations of 0.5, 1, and 2 years. The study pursued four primary objectives: (1) to generate a high‐quality, *de novo* transcriptome assembly specific to *C. bowringi* ; (2) to identify differentially expressed genes (DEGs) associated with both diapause duration and sexual dimorphism; (3) to conduct rigorous functional enrichment analyses—including Gene Ontology (GO) and Kyoto Encyclopedia of Genes and Genomes (KEGG) pathway analyses—to elucidate duration‐dependent and sex‐biased biological processes and molecular pathways; and (4) to propose a preliminary, integrative model of the core molecular architecture underpinning long‐term diapause, with explicit attention to sex‐specific regulatory mechanisms. By jointly interrogating duration progression and sexual divergence, this work advances a mechanistic understanding of extended dormancy in insects and establishes a foundational genomic resource for studies on adaptive dormancy strategies, thereby informing both evolutionary biology research and the development of ecologically informed pest management interventions.

## 2 Materials and Methods

### 2.1 Insect Source, Diapause Induction and Sample Collection

Adults of *C. bowringi* were collected from leaf surfaces in vegetable fields in Xiushui County, Jiangxi Province, People's Republic of China (29°1′N, 114°4′E) in April 2023, April 2024, and November 2024. Following collection, beetles were transferred to large glass containers (diameter: 18 cm; height: 32 cm) containing moistened soil where they spontaneously burrowed to initiate reproductive diapause. The date of entry into diapause was recorded for each cohort. Containers were maintained under semi‐natural outdoor conditions at Jiangxi Agricultural University, with exposure to ambient temperature and natural photoperiod. All adults emerged synchronously from the soil in March 2025. Accordingly, individuals that entered diapause in April 2023, April 2024, and November 2024 represent 2‐year, 1‐year, and 0.5‐year diapause durations, respectively (Figure S1). Notably, in our study region, mean daily temperatures in both April and November are consistently ≤ 20°C; at these temperatures, all *C. bowringi* adults enter diapause regardless of photoperiod (Xue et al. 2002), confirming that both summer‐entry (April) and winter‐entry (November) cohorts were induced by the same low‐temperature trigger.

To elucidate molecular adaptations associated with extended diapause, adults exhibiting 0.5 years of diapause were designated as control groups, providing a physiological and transcriptional baseline for comparative analysis against those experiencing prolonged dormancy (1‐ and 2‐year diapause). The experimental design comprised six treatment groups, stratified by both diapause duration and sex, with three independent biological replicates per group. Each replicate consisted of five individuals of the same sex and diapause duration pooled to obtain sufficient RNA. A total of 90 individuals (6 groups × 3 replicates × 5 individuals) were used for RNA‐seq analysis. When the adults with different diapause durations emerged from the soil, they were immediately frozen in liquid nitrogen and stored at −80°C until RNA extraction.

### 2.2 Total RNA Extraction, Library Construction, and Sequencing

Total RNA was extracted from each sample using TRIzol Reagent (Invitrogen, USA). RNA purity and concentration were quantified spectrophotometrically using the NanoDrop 2000 system, and RNA integrity was rigorously assessed via capillary electrophoresis on the Agilent 2100 Bioanalyzer; only samples with an RNA Integrity Number (RIN) ≥ 8.0 were retained for downstream analysis. Library construction was performed using the NEBNext Ultra RNA Library Prep Kit for Illumina (New England Biolabs, USA), following the manufacturer's protocol. Specifically, polyadenylated [poly(A)+] mRNA was selectively enriched using oligo(dT)‐conjugated magnetic beads; the enriched mRNA was then fragmented in a controlled manner using a proprietary fragmentation buffer, followed by first‐strand cDNA synthesis with reverse transcriptase and second‐strand cDNA synthesis. The resulting double‐stranded cDNA underwent end repair, 3′‐adenylation, and ligation of dual‐indexed Illumina‐compatible adapters. Adapter‐ligated fragments were size‐selected by magnetic bead‐based purification to yield inserts of optimal length (approximately 200–500 bp), and libraries were amplified by PCR under optimized cycling conditions. Final libraries were validated for concentration, fragment size distribution, and adapter dimer contamination prior to sequencing on an Illumina HiSeq platform in paired‐end mode with 150‐base reads (PE150).

### 2.3 Bioinformatics Analysis Workflow

#### 2.3.1 Data Quality Control and Sequence Assembly

Raw sequencing reads underwent stringent quality control to eliminate: (i) reads containing adapter sequences; (ii) low‐quality reads—defined as those in which > 50% of nucleotide positions exhibited a Phred quality score ≤ 10; and (iii) reads with > 10% ambiguous bases (N content). Quality control was performed using in‐house Perl scripts. The resulting high‐quality, adapter‐ and artifact‐free reads constituted the clean dataset used for all downstream analyses. Subsequently, clean reads from all samples were jointly assembled *de novo* using Trinity (r20131110) (Grabherr et al. 2011), a widely adopted, transcriptome‐optimized assembler designed to enhance reconstruction fidelity—particularly for low‐abundance and alternatively spliced transcripts. Assembly parameters were set as follows: minimum contig length = 200 bp; maximum paired‐end distance for contig clustering = 500 bp.

To remove potential contamination from non‐arthropod sources (e.g., endosymbionts, soil microorganisms, or human‐derived sequences), we performed a decontamination step at the assembled transcript level. All Unigene sequences were aligned against the NCBI NT database using BLASTn (*E*‐value ≤ 1e‐5) (Altschul et al. 1990; Camacho et al. 2009), and transcripts with best hits to non‐arthropod organisms (including bacteria, fungi, plants, and human sequences) were removed; taxonomy was assigned from the BLAST+ output using the associated taxonomy utilities (Camacho et al. 2009). This step eliminated 16,132 Unigenes (24.3% of the initial assembly; 16,097 with best hits to Bacteria and 35 to Fungi), resulting in a final set of 50,247 clean Unigenes used for all downstream analyses. Assembly contiguity was assessed by calculating the N50 length of unigenes and by BUSCO (v5.2.0) analysis against the insecta_odb10 database.

#### 2.3.2 Gene Function Annotation and Expression Quantification

The assembled unigene sequences were annotated by sequence similarity searches against multiple public databases: the NCBI Non‐Redundant (NR) Protein Database (accessed March 2025), Swiss‐Prot (March 2025), COG (Clusters of Orthologous Groups, March 2025), KOG (Eukaryotic Orthologous Groups, March 2025), Pfam (Protein Family, March 2025), Gene Ontology (GO, March 2025), and KEGG (Kyoto Encyclopedia of Genes and Genomes, Release 107.0), and eggNOG (v4.5, accessed March 2025). Homology‐based annotation was performed using DIAMOND (v2.0.15) (Buchfink et al. 2015) with an *E*‐value threshold of ≤ 1 × 10^−5^. HMMER (v3.3.2) (Eddy 1998) was used for protein domain identification against the Pfam database as a complementary approach to homology‐based BLAST searches. While DIAMOND identifies homologs based on sequence similarity, HMMER employs profile hidden Markov models to detect evolutionarily conserved protein domains even in cases of low overall sequence similarity, thereby improving annotation sensitivity for divergent or poorly characterized sequences, with a stringent *E*‐value cutoff of ≤ 1 × 10^−10^. Subsequently, high‐quality (clean) reads from each sample were aligned to the unigene reference assembly using Bowtie2 (v2.5.0) (Langmead et al. 2009), and transcript abundance was estimated using RSEM (v1.3.3) (Dewey and Bo 2011). Gene expression levels were quantified as FPKM (Fragments Per Kilobase of transcript per Million mapped reads).

#### 2.3.3 Differential Expression Analysis and Functional Enrichment

Differential expression analysis was conducted between experimental groups using raw read counts as input for DESeq2 (v1.42.0, R v4.3.2) (Love et al. 2014), with the model ~ Sex + Duration + Sex:Duration. Gene‐level counts were chosen over transcript‐level counts because isoform‐level assembly in a *de novo* transcriptome can be incomplete, and gene‐level aggregation provides more robust estimates for differential expression analysis in non‐model species. Differentially expressed genes (DEGs) were defined as those exhibiting an absolute log_2_ fold change ≥ 1 and an adjusted *p*‐value (Benjamini–Hochberg false discovery rate, FDR) < 0.01. Nine pairwise comparisons were performed: F0.5 versus M0.5, F0.5 versus F1.0, F0.5 versus F2.0, M0.5 versus M1.0, M0.5 versus M2.0, F1.0 versus F2.0, M1.0 versus M2.0, F1.0 versus M1.0, and F2.0 versus M2.0 (where F = female, M = male, and numbers indicate diapause duration in years). In addition, to formally test the Sex × Duration interaction, we performed a likelihood ratio test (LRT) comparing the full model with a reduced model without the interaction term; the statistic, 2 × (log‐likelihood of the full model −log‐likelihood of the reduced model), is non‐negative by construction and was assessed against a *χ* ^2^ distribution with one degree of freedom. For a small number of genes the dispersion‐fitting optimizer returned a transiently negative statistic, which was truncated to zero before *p*‐value computation; the values reported in Table S5 have been corrected accordingly. Subsequent functional enrichment analyses—including GO term enrichment and Kyoto Encyclopedia of Genes and Genomes (KEGG) pathway enrichment—were performed on the DEG set. Statistical significance of enrichment was determined via the hypergeometric test, with results considered significant at an FDR‐adjusted threshold of < 0.05. Enrichment results were visualized using ClusterProfiler (v4.10.0) (Yu et al. 2012) and other standard bioinformatics visualization tools.

## 3 Results

### 3.1 Quality Assessment and *De Novo* Transcriptome Assembly

The 18 libraries yielded 122.96 Gb of high‐quality clean reads (8.20 × 10^8^ reads), with ≥ 5.72 Gb per sample. Q30 exceeded 94.25% in all samples and GC content ranged between 38.38% and 41.46% (Table 1), satisfying established standards for *de novo* transcriptome assembly and supporting robust downstream analyses.

**TABLE 1:** Summary of RNA‐seq metrics from *C. bowringi* transcriptomes.

Group	Read number	Base number	GC content (%)	≥ Q30 (%)
F0.5	23,584,478 ± 1,250,224	7,027,782,079 ± 389,449,264	41.18 ± 0.26	95.76 ± 0.66
M0.5	24,629,605 ± 2,073,306	7,338,663,202 ± 615,259,435	39.13 ± 0.62	95.71 ± 0.56
F1.0	23,387,095 ± 1,329,869	6,977,131,911 ± 398,301,768	40.59 ± 0.15	95.75 ± 0.60
M1.0	21,994,790 ± 1,886,812	65,672,143,434 ± 565,932,719	40.88 ± 0.90	95.55 ± 0.54
F2.0	21,202,820 ± 724,978	6,327,635,997 ± 212,952,003	41.13 ± 0.29	95.43 ± 0.78
M2.0	22,614,578 ± 1,222,126	6,746,774,004 ± 360,277,790	40.48 ± 0.34	95.51 ± 0.21

*Note:* Data are presented as mean ± SD based on clean reads.

The initial assembly comprised 66,379 Unigenes, with a total length of 55,438,785 bp, an N50 of 1598 bp, a mean length of 835.19 bp, and 14,680 Unigenes (22.12%) exceeding 1 kb (Table S1). Of these, 16,132 Unigenes (24.3%) had best BLASTn hits to non‐arthropod organisms (16,097 to Bacteria and 35 to Fungi) and were removed as contamination, leaving 50,247 clean Unigenes with a total length of 45,728,806 bp, an N50 of 1886 bp, a mean length of 910.08 bp, a GC content of 37.79%, and 12,509 Unigenes (24.90%) exceeding 1 kb (Table S1). The removed fraction was markedly shorter than the retained set (mean length 601.91 bp, N50 759 bp) and had a strongly elevated GC content (55.18% vs. 37.79%), a compositional signature typical of bacterial sequences, so the filtering step improved both the contiguity and the compositional homogeneity of the assembly. BUSCO analysis against the insecta_odb10 database showed C: 94.9%, F: 1.8%, M: 3.3% (Table S2), confirming the high completeness of the assembly. This high‐fidelity transcriptome serves as a reliable reference for downstream gene annotation and differential expression analysis in *C. bowringi* .

### 3.2 Unigene Functional Annotation

Annotation was performed on the 50,247 decontaminated Unigenes, of which 30,243 (60.2%) received at least one functional annotation—constituting a robust functional resource for downstream analyses. Annotation distribution across databases is summarized in Figure 1 and Table S2. Pfam yielded the highest number of annotations (23,753 Unigenes), reflecting its strength in identifying evolutionarily conserved protein domains. GO followed with 19,158 annotated Unigenes, providing standardized, ontology‐based functional categorization (biological process, molecular function, and cellular component). NR contributed 15,253 annotations, offering broad coverage of protein sequence homology. Notably, among the annotated Unigenes, 11,934 (39.5% of the annotated subset) exhibited transcript lengths exceeding 1000 bp—suggesting that longer transcripts, which are more likely to represent full‐length or near‐full‐length coding sequences, demonstrate higher annotation success rates.

**FIGURE 1:**
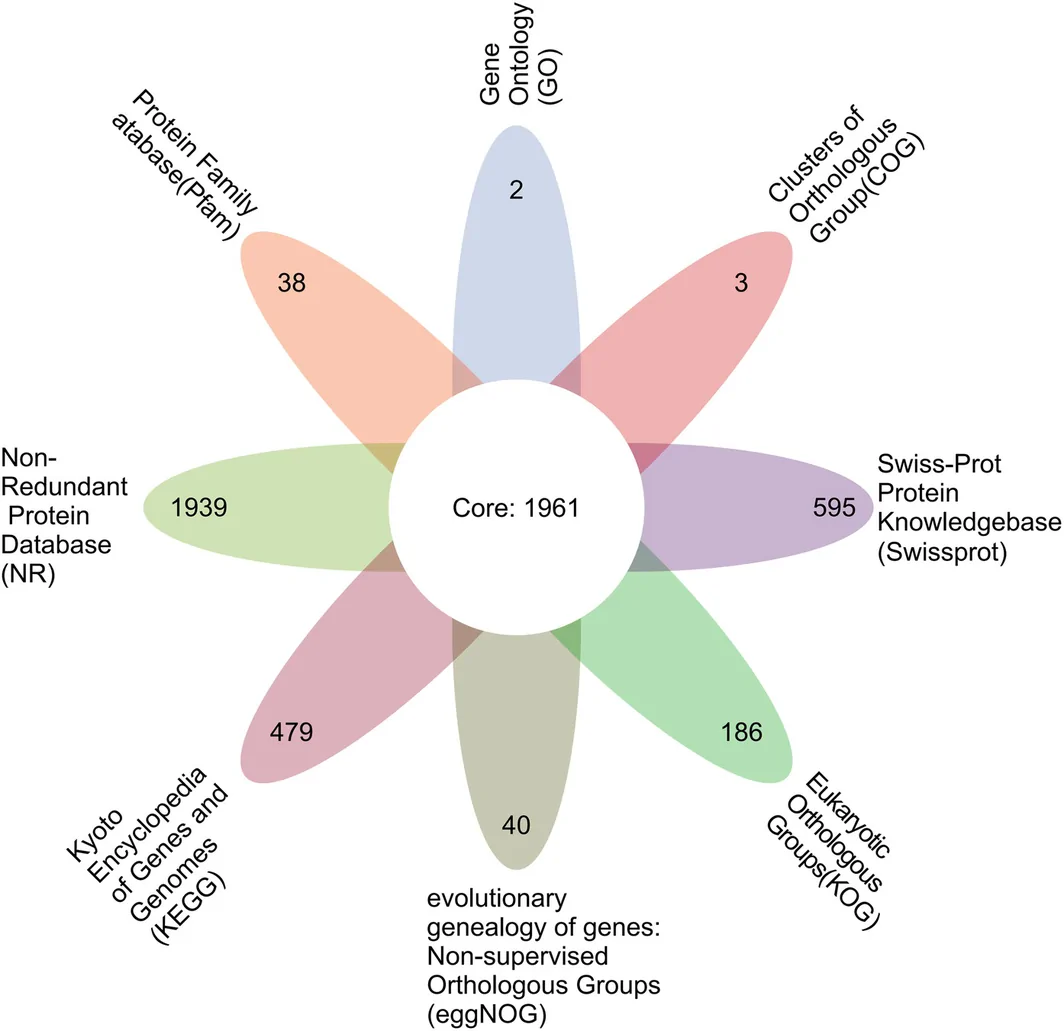
The number of identified unigenes based on the selected databases. The term “Core” has been defined as Unigenes that received at least one annotation across any of the queried databases.

To characterize the assembled transcriptome, we first performed functional annotation of the identified genes using GO, COG, and KEGG databases, with the overall annotation results summarized in Figure 2. Within the GO framework (Figure 2A), the five most highly represented categories—based on the number of annotated genes—were: (i) cellular process, (ii) binding, (iii) metabolic process, (iv) cellular anatomical entity, and (v) catalytic activity. In the COG classification (Figure 2B), the top five functional categories were: (i) translation, ribosomal structure and biogenesis; (ii) general function prediction only; (iii) transcription; (iv) signal transduction mechanisms; and (v) posttranslational modification, protein turnover, and chaperones. Functional classification of annotated genes revealed that the predominant KEGG pathway categories were: (i) metabolism; (ii) transport and catabolism; (iii) signal transduction; (iv) amino acid metabolism; and (v) carbohydrate metabolism (Figure 2C). Collectively, these functional distribution patterns align closely with those observed in transcriptomic studies of other insect species, underscoring the evolutionary conservation of fundamental biological processes. Moreover, the identification of a substantial number of previously uncharacterized genes significantly expands the current *C. bowringi* transcriptome resource and provides a robust foundation for downstream functional characterization.

**FIGURE 2:**
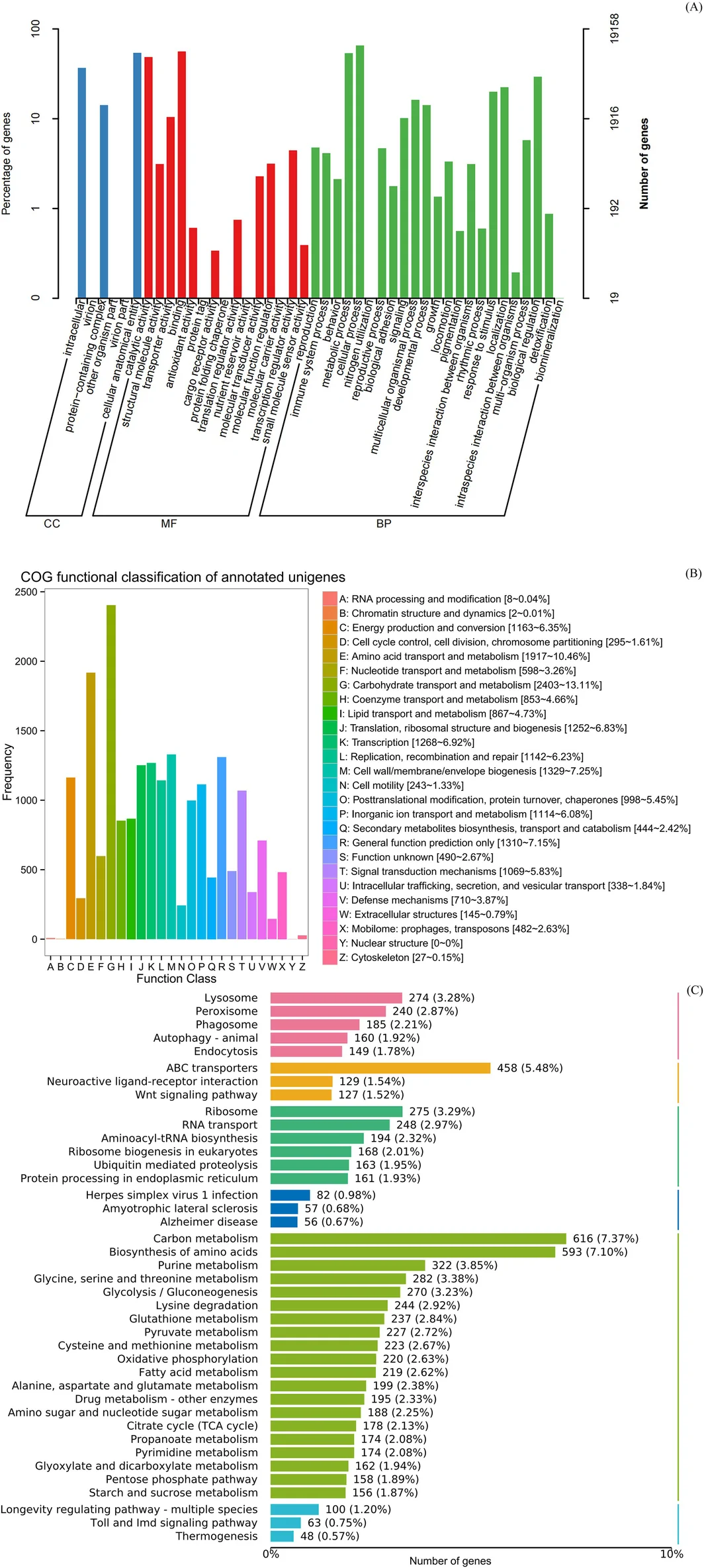
Functional annotation results of identified unigenes. (A) Gene Ontology annotation that successfully annotated genes will group into three main GO domains: biological process (BP), cellular component (CC), molecular function (MF). (B) Clusters of Orthologous Groups (COG) classification of putative proteins. (C) Kyoto Encyclopedia of Genes and Genome (KEGG) pathway contains: metabolism, genetic information processing, environmental information processing, cellularprocesses, organismal systems and human diseases.

### 3.3 Global Analysis of Differentially Expressed Genes

A total of nine pairwise comparisons were performed, revealing substantial variation in the number of differentially expressed genes (DEGs), consistent with sex‐ and duration‐dependent transcriptional reprogramming (Table 2). The largest DEG count—3825—was observed in the comparison between males and females following 0.5‐year diapause (F0.5 vs. M0.5), indicating pronounced sexual dimorphism at this life stage. In matched duration comparisons within each sex, females yielded equal or greater numbers of DEGs (F0.5 vs. F1.0: 389; F0.5 vs. F2.0: 284) than males (M0.5 vs. M1.0: 262; M0.5 vs. M2.0: 279), so the sexes differ not in overall transcriptional responsiveness but in the functional composition and directionality of their duration‐associated responses (Figure 3; Section 3.4). To assess whether this difference could be technical, we compared sequencing depth and dispersion estimates between the sexes (Table S4). Total mapped reads were closely matched (females: 19.89 ± 1.14 M; males: 20.13 ± 1.88 M), whereas gene‐wise median dispersion was higher in males (8.755, IQR 9.598) than in females (2.826, IQR 9.799). Higher dispersion in males would be expected to reduce rather than inflate DEG detection in that group, but the ~3‐fold difference in median dispersion is not negligible and sex‐specific dispersion may contribute to the DEG counts reported here; we therefore base our conclusions on the functional composition of the sex‐specific DEG sets rather than on DEG counts alone. Consistent with the high annotation coverage (Table 3), KEGG enrichment analysis of these DEG sets (detailed in Section 3.4) identified glutathione metabolism, autophagy, and lysosomal pathways as the most consistently enriched categories across both sex and duration comparisons, underscoring their core roles in cytoprotection during prolonged diapause.

**TABLE 2:** Number of differentially expressed genes across comparison groups.

Comparison	All DEGs	Increased transcript abundance	Decreased transcript abundance
F0.5 versus M0.5	3825	2349	1476
F0.5 versus F1.0	389	270	119
F0.5 versus F2.0	284	181	103
F1.0 versus M1.0	2585	1797	788
M0.5 versus M1.0	262	128	134
M0.5 versus M2.0	279	76	203
F2.0 versus M2.0	2282	1645	637

*Note:* DEGs were defined as those with |log_2_(Fold Change)| ≥ 1 and FDR < 0.01.

**FIGURE 3:**
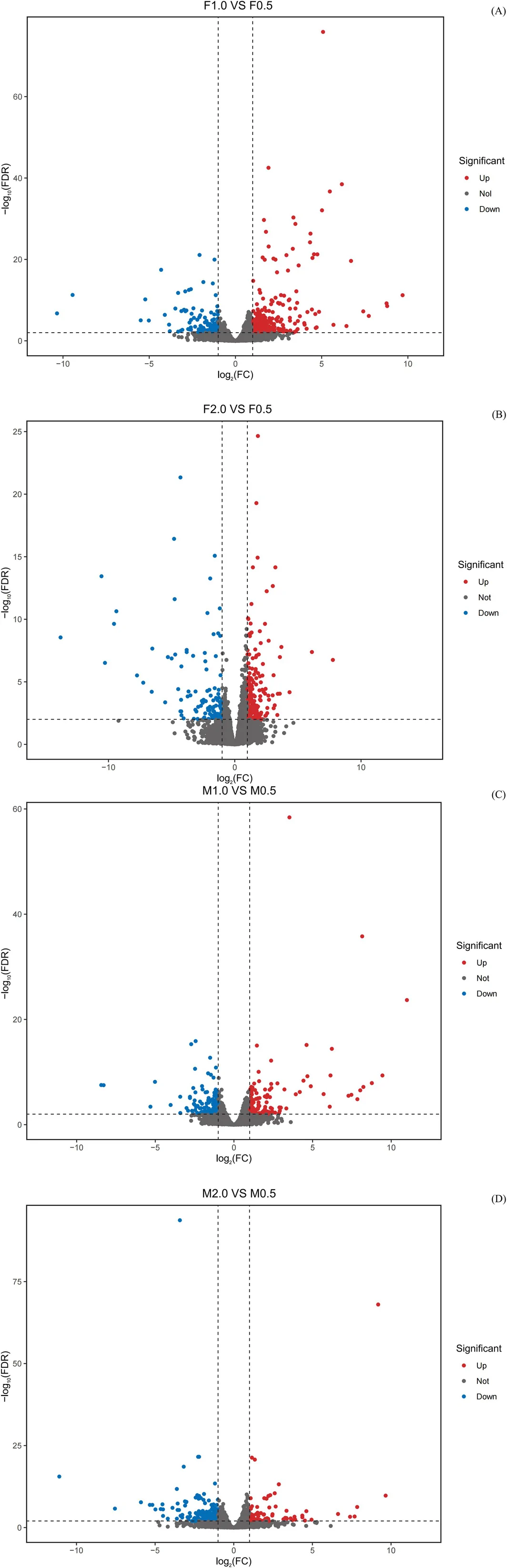
Volcano plots depicting differentially expressed genes associated with diapause duration across sexes. The *x*‐axis represents the log_2_‐transformed fold change in gene expression (log_2_FC), and the *y*‐axis denotes –log_10_(FDR), reflecting the statistical significance of differential expression (FDR: False discovery rate, Benjamini–Hochberg adjusted). Red dots indicate genes with increased transcript abundance in the comparison group relative to the control; blue dots indicate genes with decreased transcript abundance; gray dots represent genes with no significant differential expression (|log_2_FC| < 1 or FDR ≥ 0.01). Subpanels depict pairwise comparisons: (A) F1.0 versus F0.5 (1‐year females vs. 0.5‐year females, control); (B) F2.0 versus F0.5 (2‐year females vs. 0.5‐year females, control); (C) M1.0 versus M0.5 (1‐year males vs. 0.5‐year males, control); (D) M2.0 versus M0.5 (2‐year males vs. 0.5‐year males, control).

**TABLE 3:** Number of annotated DEGs across functional databases.

Comparison	Total	COG	GO	KEGG	KOG	NR	Pfam	Swiss‐Prot	eggNOG
F0.5 versus M0.5	2741	785	2320	2270	1854	2263	2517	1252	2153
F0.5 versus F1.0	269	83	212	216	166	210	252	102	199
F0.5 versus F2.0	192	68	158	165	129	158	178	69	150
F1.0 versus M1.0	1857	527	1540	1507	1185	1509	1689	789	1434
M0.5 versus M1.0	164	58	132	138	106	136	144	60	124
M0.5 versus M2.0	177	48	137	135	109	141	164	57	130
F2.0 versus M2.0	1694	473	1420	1376	1101	1393	1529	754	1334

Notably, the proportion of genes with decreased transcript abundance increased with prolonged diapause, reaching predominance after 2 years. Hierarchical clustering of DEGs demonstrated that female samples clustered together separately from male samples, with 1‐year and 2‐year female samples forming a distinct subcluster, indicating greater transcriptional similarity among female samples across diapause durations. In contrast, male samples showed greater dispersion, with 1‐year and 2‐year male samples separated more distinctly, reflecting more pronounced duration‐dependent transcriptional remodeling in males (Figure S2). These patterns underscore duration‐resolved, stage‐specific transcriptional remodeling associated with diapause progression.

### 3.4 Functional Enrichment Analysis of Transcriptomic Changes Associated With Diapause Duration

To investigate the functional implications of the transcriptional alterations identified in Section 3.3, GO and Kyoto Encyclopedia of Genes and Genomes (KEGG) enrichment analyses were conducted on differentially expressed genes (DEGs) associated with extended diapause duration—specifically, comparisons between the 0.5‐year control group and groups exhibiting longer diapause durations, stratified by sex.

#### 3.4.1 GO Enrichment Analysis

In females, Gene Ontology (GO) enrichment analysis of differentially expressed genes (DEGs) derived from the F0.5 versus F1.0 and F0.5 versus F2.0 comparisons revealed persistent enrichment in fundamental cellular and metabolic functions—including “cellular process,” “metabolic process,” and “catalytic activity.” (Figure 4). A temporally coordinated reorganization of functional annotation was observed: the transition into 1‐year diapause was associated with significant enrichment in regulatory biological activities—specifically “biological regulation,” “signaling,” and “immune system process.” In contrast, the 2‐year comparison (F0.5 vs. F2.0) exhibited a marked functional shift toward stress resilience mechanisms, with robust enrichment in “antioxidant activity” (5 DEGs), “detoxification” (6 DEGs), and “response to stimulus” (105 DEGs). Notably, “reproductive process” remained significantly enriched at both time points, suggesting sustained transcript abundance of genes associated with reproductive function (Figure 4B).

**FIGURE 4:**
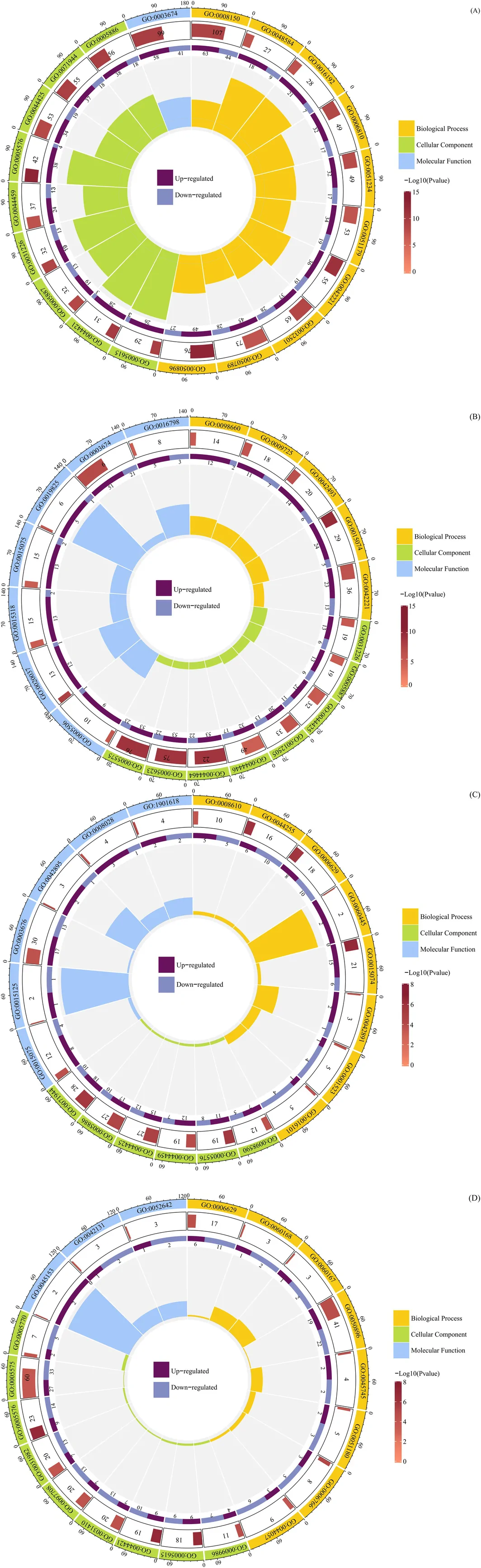
Enrichment circles of the top 20 GO terms in differentially expressed genes (DEGs). First circle: Top 20 enriched GO terms, with gene counts displayed outside the circle. Different colors represent different ontologies; Second circle: Number of differentially expressed genes (DEGs) for each GO term and the corresponding *Q*‐value. Longer bars indicate more genes, and redder colors indicate smaller *Q*‐values; Third circle: Bar plot showing the proportion of increased and decreased transcript abundance genes. Dark purple represents the proportion of increased transcript abundance genes; light purple represents the proportion of decreased transcript abundance genes; specific values are shown below the bars. Fourth circle: Rich factor value for each GO term. Subpanels depict pairwise comparisons: (A) 1‐year diapause duration females versus 0.5‐year diapause duration females (control); (B) 2‐year diapause duration females versus 0.5‐year diapause duration females (control); (C) 1‐year diapause duration males versus 0.5‐year diapause duration males (control); (D) 2‐year diapause duration males versus 0.5‐year diapause duration males (control).

In males, GO enrichment profiles demonstrated a duration‐dependent amplification of both regulatory and stress‐responsive pathways. Although core cellular processes remained consistently enriched across comparisons, functional categories such as “transporter activity,” “molecular transducer activity,” and “transcription regulator activity” showed pronounced increases in enrichment magnitude in the 2‐year comparison (M0.5 vs. M2.0) (Figure 4D). Similarly, stress‐associated terms—including “immune system process” (37 DEGs) and “response to stimulus” (195 DEGs)—exhibited substantially stronger statistical support after 2 years of dormancy. Furthermore, reproduction‐related GO terms were significantly enriched in M0.5 versus M2.0 (74 DEGs), indicating active transcriptional engagement in reproductive preparation during prolonged diapause.

#### 3.4.2 KEGG Pathway Enrichment Analysis

KEGG pathway enrichment analysis further elucidated the metabolic and signaling pathways modulated by diapause duration, revealing pronounced sex‐specific and time‐dependent patterns (Figure 5). In females, differentially expressed genes (DEGs) identified in the 1‐year diapause comparison (F0.5 vs. F1.0) were significantly enriched in pathways associated with cellular structural integrity and innate immunity—including Phagosome (ko04145; 8 DEGs), ECM–receptor interaction (ko04512; 5 DEGs), and Lysosome (ko04142; 13 DEGs) (Figure 5A). Upon extension to 2 years of diapause (F0.5 vs. F2.0), the enrichment profile shifted markedly: immune‐related signaling pathways—including the Toll‐like receptor signaling pathway (ko04620) and RIG‐I‐like receptor signaling pathway (ko04622)—were specifically enriched, alongside significant enrichment of the Longevity regulating pathway (ko04213) (Figure 5B).

**FIGURE 5:**
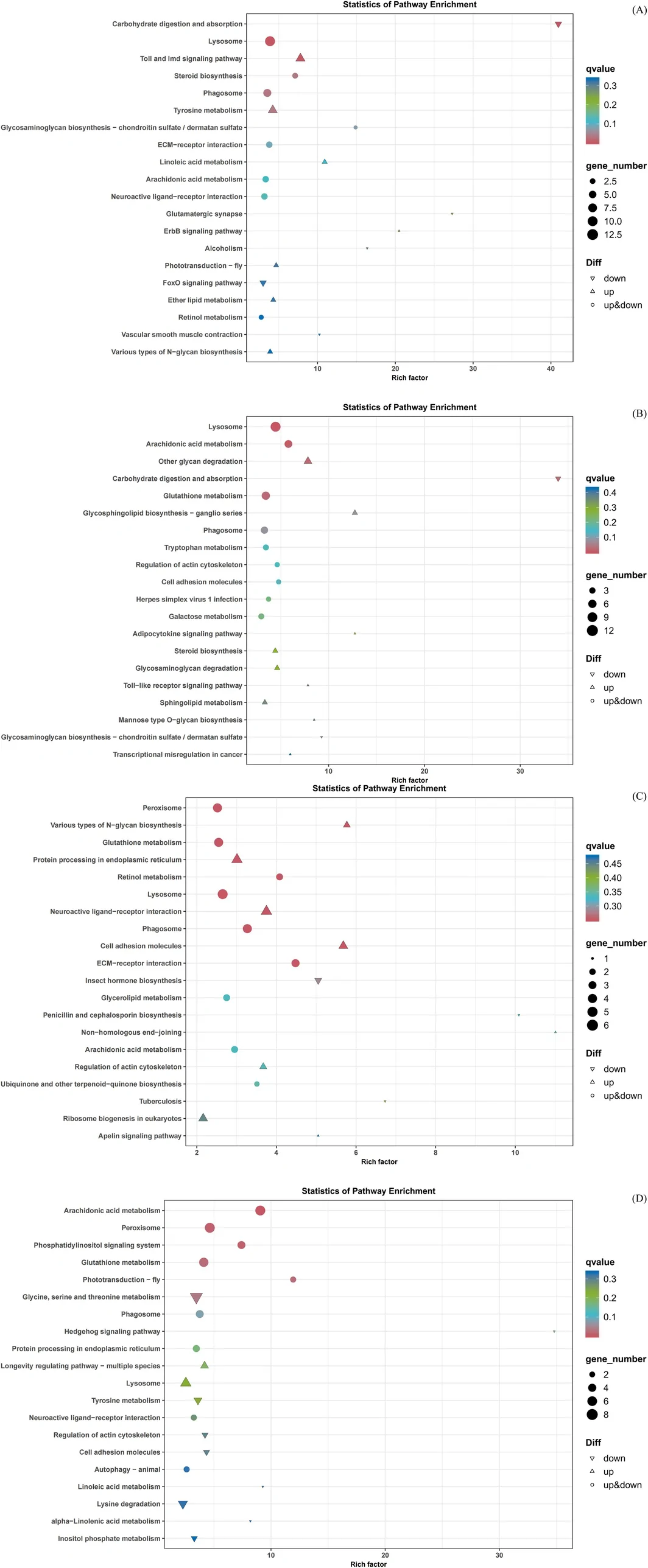
KEGG pathway enrichment scatter plots of differentially expressed genes (DEGs). The *x*‐axis represents the Rich Factor, defined as the ratio of the number of DEGs annotated to a given pathway to the total number of all genes annotated to that pathway in the background dataset (Rich Factor = DEGs in pathway/Total genes in pathway). The *y*‐axis lists the KEGG pathway names. Each dot represents a KEGG pathway. The size of each dot indicates the number of DEGs enriched in that pathway (larger dots = more DEGs). The color of each dot represents the *q*‐value (Benjamini‐Hochberg adjusted *p*‐value) for the enrichment, with red indicating higher significance (smaller *q*‐value) and blue indicating lower significance. Only the top 20 significantly enriched pathways (*q* < 0.05) are shown for each comparison. Subpanels depict pairwise comparisons: (A) F10 versus F05 (female, 1‐year vs. 0.5‐year diapause); (B) F20 versus F05 (female, 2‐year vs. 0.5‐year); (C) M10 versus M05 (male, 1‐year vs. 0.5‐year); (D) M20 versus M05 (male, 2‐year vs. 0.5‐year).

In males, DEGs from the 1‐year diapause comparison (M0.5 vs. M1.0) were predominantly enriched in homeostatic maintenance pathways—namely, Lysosome (ko04142; 6 DEGs), Phagosome (ko04145; 5 DEGs), and Protein processing in endoplasmic reticulum (ko04141; 4 DEGs) (Figure 5C). At the 2‐year time point (M0.5 vs. M2.0), enrichment reoriented toward lipid metabolism (e.g., Glycerophospholipid metabolism, ko00564), oxidative and xenobiotic stress response (e.g., Glutathione metabolism, ko00480; 7 DEGs), and developmental signaling pathways potentially linked to post‐diapause reactivation readiness (e.g., Wnt signaling pathway, ko04310) (Figure 5D).

A detailed list of the genes driving each of these key pathways, with Unigene IDs, gene names (where available), log_2_ fold changes and FDR‐adjusted *p* values, is provided in Table S3. For example, glutathione metabolism (ko00480) was enriched in M0.5 versus M2.0 through opposing responses among glutathione peroxidase paralogues, one increasing (TRINITY_DN6164_c1_g1, log_2_FC = +4.615, FDR = 1.02 × 10^−5^) and another decreasing (TRINITY_DN12400_c0_g1, log_2_FC = −3.896, FDR = 9.90 × 10^−7^), whereas glutathione S‐transferase increased in females (F0.5 vs. F2.0; TRINITY_DN126_c0_g1, log_2_FC = +1.031, FDR = 3.46 × 10^−4^). These genes and their fold changes are interpreted in Section 4.1.

Notably, several core pathways implicated in cytoprotection and cellular homeostasis—including Glutathione metabolism (ko00480), Autophagy—animal (ko04140), and Lysosome (ko04142)—exhibited consistent enrichment across the majority of pairwise comparisons, highlighting their essential and sustained contribution to prolonged dormancy. Moreover, Arachidonic acid metabolism (ko00590) was robustly enriched in both prolonged female diapause (F0.5 vs. F2.0; 7 DEGs) and 2‐year male diapause (M0.5 vs. M2.0; 8 DEGs), suggesting a conserved role in regulating inflammatory and immune‐modulatory processes during extended quiescence.

## 4 Discussion

This study presents the first multi‐duration, sex‐stratified transcriptomic analysis of prolonged diapause in *C. bowringi* , uncovering a highly dynamic molecular landscape marked by duration‐dependent transcriptional reprogramming and pronounced sexual dimorphism. Our results corroborate and substantially extend the prevailing conceptualization of insect diapause—as a genetically orchestrated, metabolically active physiological state—rather than a passive state of quiescence (Denlinger 2002; MacRae 2010; Easwaran and Montell 2023).

### 4.1 Core Adaptive Strategy: Patterns Consistent With Metabolic Suppression and Cellular Protection

A central adaptive mechanism underpinning prolonged diapause in *C. bowringi* is the coordinated regulation of metabolic suppression and cellular maintenance. Transcriptomic and functional analyses reveal patterns consistent with systemic downregulation of energy metabolism pathways—particularly oxidative phosphorylation, glycolysis, and mitochondrial ATP synthesis—which collectively are associated with a state of profound metabolic depression. This response aligns with the canonical physiological hallmark of diapause and serves to conserve finite endogenous energy reserves under extended nutrient limitation (Hahn and Denlinger 2011). Concomitantly, multiple cytoprotective pathways show robust and persistent increased transcript abundance, including glutathione‐mediated redox homeostasis, selective autophagy, lysosomal biogenesis and function, and Phase I/II detoxification enzymes. Such coordinated enhancement is consistent with a pattern of resource allocation toward macromolecular integrity, damage surveillance, and stress resilience. This dual‐regulatory architecture supports long‐term dormancy by simultaneously minimizing energetic demand and maximizing cellular fidelity—thereby mitigating cumulative oxidative damage, proteotoxic stress, and opportunistic microbial threats (Hand et al. 2016). The consistent enrichment of these pathways across all sampled diapause durations (ranging from 6 months to > 24 months) indicates their constitutive role in the diapause program of *C. bowringi* . Critically, the observed intraspecific variation in diapause duration—spanning several‐fold differences among genetically similar individuals—likely constitutes an evolutionary bet‐hedging strategy that enhances population‐level persistence in the face of spatiotemporal environmental uncertainty (Soula and Menu 2003). Similar metabolic suppression and cytoprotection are observed in other aestivating insects. In *Psylliodes chrysocephala*, summer diapause involves reduced CO_2_ emission, glycogen depletion, and preferential lipid storage (Güney et al. 2024), accompanied by global downregulation of translation‐related proteins (Güney et al. 2026). In *Helicoverpa assulta* , high‐temperature‐induced diapause leads to increased energy reserves and persistently low respiration (Liu et al. 2016). Compared to these relatively short aestivation periods, the multi‐year diapause in *C. bowringi* features sustained activation of glutathione metabolism and autophagy, indicating additional protective layers required for extended dormancy.

A gene‐level view qualifies this pathway‐level pattern in two respects. Within glutathione metabolism (ko00480), a single peroxidase paralogue increased in both the female 2‐year and male 1‐year comparisons (TRINITY_DN2991_c0_g1, log_2_FC = +1.478 and +1.532, FDR < 0.001), whereas other peroxidase paralogues moved in the opposite direction in males at 2 years (TRINITY_DN6164_c1_g1, +4.615; TRINITY_DN6438_c0_g1, −1.694; TRINITY_DN12400_c0_g1, −3.896), while a glutathione S‐transferase and a glutathione hydrolase increased in females (TRINITY_DN126_c0_g1, +1.031; TRINITY_DN6833_c0_g1, +3.606). Pathway enrichment therefore summarizes a mixed, paralogue‐specific response rather than a uniform induction—a distinction that is invisible at the pathway level and that cautions against reading the result as straightforward activation of antioxidant defense. The lysosomal and autophagic axis behaves similarly: its signal is carried mainly by cathepsins (cathepsin L, TRINITY_DN11333_c0_g1, +9.657, FDR = 1.82 × 10^−10^ in males at 2 years; TRINITY_DN20720_c0_g1, +1.133; and in females cathepsin L1‐like TRINITY_DN20916_c0_g1, +1.682 and cathepsin B TRINITY_DN4709_c0_g1, +1.241) and by lysosomal acid phosphatase (TRINITY_DN20010_c0_g1, +1.648), but CD63 (TRINITY_DN14403_c0_g1, −9.374, FDR = 2.29 × 10^−11^) and one cathepsin L1‐like transcript (TRINITY_DN14612_c0_g1, −13.79) declined sharply, indicating that the compartment is remodeled rather than globally amplified. Cross‐species comparison is informative here: the strong cathepsin and acid‐phosphatase signal in *C. bowringi* contrasts with the weaker and partly divergent proteolytic signature reported for aestivating *P. chrysocephala* (Güney et al. 2024, 2026), whereas diapause maintenance in *L. decemlineata* likewise involves coordinated lipid and proteostasis remodeling (Cedden et al. 2025). The amplitude and paralogue composition of these modules thus appear to scale with diapause duration and life‐history context rather than being fixed features of the diapause program.

A further essential component of this cytoprotective network is trehalose, the principal hemolymph sugar in insects, which acts as both a rapidly mobilizable energy source and a chemical chaperone against heat and desiccation (Elbein et al. 2003). Recent functional studies in related leaf beetles demonstrate that trehalose transporters (TRETs) regulate spatiotemporal trehalose distribution during diapause: in *Psylliodes chrysocephala*, TRET‐1 exports trehalose from the fat body to hemolymph while TRET‐2 imports it into Malpighian tubules, and knockdown of TRET‐2 impairs heat tolerance (Güney et al. 2025); in *C. bowringi* , silencing TRET1a elevates fat body trehalose and suppresses stress‐related genes, whereas TRET1b is ovary‐specific in non‐diapause females (Li et al. 2020). Our data do indicate duration‐associated changes in this pathway, but contigs annotated as trehalose transporters were largely fragmented and did not consistently pass our differential‐expression thresholds, so we treat the transporter evidence as hypothesis‐generating only. Given the partly sex‐specific roles of TRET1a and TRET1b in this species (Li et al. 2020) and the tissue partitioning documented in *P. chrysocephala* (Güney et al. 2025), these patterns remain consistent with coordinated trehalose flux contributing to the strategy outlined above, but whole‐body transcript data cannot resolve the tissue‐specific partitioning that determines its physiological function; tissue‐resolved TRET quantification together with trehalose titer measurements is therefore needed to test it.

### 4.2 Sex‐Specific Diapause Regulation: Divergent Life‐History Trade‐Offs

The sexually dimorphic transcriptional dynamics observed during diapause are consistent with sex‐specific life‐history strategies. Females yielded equal or greater numbers of DEGs than males in the matched duration comparisons (389 and 284 vs. 262 and 279), and because higher male dispersion (Table S4) would tend to reduce rather than inflate DEG detection, the sex difference concerns the directionality and functional composition of the response rather than its magnitude. In females, the transcriptional changes were associated with sustained enrichment of gene ontology (GO) terms associated with reproductive processes and somatic maintenance, suggesting patterns consistent with allocation of resources toward preserving long‐term reproductive potential and cellular integrity. Such patterns align with the life‐history theory prediction that females prioritize future fecundity over immediate physiological responsiveness (Flatt and Heyland 2011). Conversely, males showed pronounced enrichment in GO categories related to signal transduction, molecular transducer activity, and physiological homeostasis—indicative of heightened regulatory plasticity aimed at sustaining functional competence and readiness for post‐diapause behaviors, including mate‐searching, which likely requires rapid physiological activation. While these findings partially parallel observations in the Colorado potato beetle—where prolonged diapause imposes sex‐differentiated fertility and fitness costs (Margus and Lindström 2020)—they further reveal a key interspecific distinction: in *C. bowringi* , the female‐biased transcriptional pattern appears to buffer against reproductive decline following extended diapause. This is consistent with prior empirical reports indicating that prolonged diapause exerts neutral or even beneficial effects on female fecundity in this species (Wang et al. 2006). Gene‐level patterns support this reading. In females, farnesyl pyrophosphate synthase increased (TRINITY_DN896_c0_g1, log_2_FC = +1.436, FDR = 7.05 × 10^−15^), consistent with continued investment in juvenile‐hormone precursor biosynthesis, whereas vitellogenin‐1‐like transcripts decreased after 1 year (TRINITY_DN2869_c0_g1, log_2_FC = −1.599), indicating that yolk‐protein provisioning is reduced rather than maintained during dormancy. Males at 2 years showed the opposite response for the same transcript (TRINITY_DN2869_c0_g1, +7.394; TRINITY_DN528_c0_g1, +7.685) together with increased abundance of endoplasmic‐reticulum chaperones (78 kDa glucose‐regulated protein TRINITY_DN20216_c0_g1, +1.287 and endoplasmin TRINITY_DN8192_c0_g1, +1.044, both M0.5 vs. M1.0; alpha‐crystallin B chain TRINITY_DN6167_c0_g1, +1.922 and TRINITY_DN7821_c0_g1, +1.236, M0.5 vs. M2.0). Two caveats qualify this interpretation: vitellogenin also carries non‐reproductive stress‐protection and immune functions, so the male increase need not reflect reproductive provisioning, and the chaperone signals derive from comparisons at different durations. Both are consistent with the sex‐specific proteostasis signatures reported for *L. decemlineata* (Cedden et al. 2025; Torson et al. 2026), and together these patterns reinforce the view that sexual dimorphism in diapause regulation is a conserved feature across chrysomelid beetles.

### 4.3 Duration‐Dependent Dynamics of Diapause: Evidence for a Structured, Duration‐Specific Regulatory Program

Our findings robustly support the conceptualization of diapause not as a metabolically quiescent or static state, but rather as a temporally structured, duration‐specific regulatory program—each duration characterized by distinct, stage‐specific molecular signatures (Koštál 2006; Roncalli et al. 2018). Although our design compares three duration endpoints rather than a true developmental time series, the consistent duration‐dependent patterns suggest that diapause progression involves sequential molecular transitions. During the first year of extended diapause, transcriptomic and functional enrichment analyses revealed pronounced activation of immune system processes, extracellular matrix remodeling pathways, and key developmental signaling cascades—collectively indicative of active, adaptive processes involving tissue homeostasis maintenance and structural reorganization. This early phase exhibits strong mechanistic parallels with diapause initiation in well‐studied model systems, including patterns consistent with suppression of mitochondrial oxidative phosphorylation and transcriptional upregulation of conserved stress‐response effectors (Lecheta et al. 2025). In contrast, the second year was marked by a functional pivot toward enhanced cellular stress defense mechanisms—specifically, heightened activity in innate immune modules and significant enrichment in evolutionarily conserved longevity‐associated pathways (e.g., insulin/IGF‐1 signaling and sirtuin‐mediated regulation). This temporal shift likely reflects progressive accumulation of macromolecular damage and cellular stress, and may reflect processes associated with eventual diapause termination. Notably, sexually dimorphic patterns emerged: in males, the transition into the second year coincided with patterns consistent with increased reliance on lipid catabolism and reactivation‐primed signaling networks—including canonical Wnt/β‐catenin pathway components—thereby highlighting time‐ and sex‐dependent physiological reprioritization. The enrichment of lipid metabolic pathways is consistent with their dual role as both a sustained energy reservoir and a critical source of signaling lipids (e.g., sphingolipids, oxylipins) essential for dormancy maintenance and stress adaptation (Sinclair and Marshall 2018; Zhang et al. 2025). Collectively, these temporally resolved molecular transitions provide a mechanistic basis for observed physiological trade‐offs—most notably, the inverse correlation between diapause duration and adult thermotolerance (Ni et al. 2025)—which may represent an energetic and resource‐allocation cost arising from prolonged metabolic depression and preferential investment in long‐term cellular defense over acute stress resilience.

### 4.4 Implications for the Molecular Regulatory Network

This study reveals a dynamic molecular landscape that significantly expands the current understanding of the diapause regulatory network in *Colaphellus bowringi*. Recent investigations in this species have established the PBAP chromatin‐remodeling complex as a critical mediator translating photoperiodic signals into juvenile hormone (JH)‐dependent induction of summer diapause (Tian et al. 2025), while circadian clock genes—including period (per) and timeless (tim)—have been shown to couple photoperiodic input with lipid accumulation during diapause preparation (Zhu et al. 2019). Complementing these findings, our transcriptomic analysis—focused specifically on the maintenance and prolongation phases of diapause—identifies core effector pathways, such as glutathione metabolism and autophagy, which are likely positioned downstream of upstream photoperiodic and endocrine signaling events. Collectively, these results support a hierarchical and functionally integrated regulatory axis: environmental photoperiod is sensed and transduced via epigenetic (e.g., PBAP), neuroendocrine (e.g., JH), and circadian mechanisms, ultimately converging on the execution of sustained physiological adaptations—including metabolic suppression and cellular stress resilience. Given the multifaceted nature of prolonged diapause, future work should explore additional regulatory layers, such as DNA methylation, histone modifications, and host–microbiome crosstalk, as proposed in recent conceptual frameworks (Denlinger 2023).

### 4.5 Limitations and Future Directions

This transcriptomic study offers a comprehensive characterization of gene expression dynamics associated with diapause; however, several methodological and interpretive limitations warrant consideration. First, the use of whole‐adult specimens for RNA extraction may obscure tissue‐specific transcriptional regulation, thereby limiting resolution of spatially restricted molecular mechanisms. Subsequent investigations employing dissected tissues—particularly metabolically and developmentally critical organs such as the fat body, gonads, and central nervous system—would enhance mechanistic precision (Poelchau et al. 2013). Second, transcript abundance does not necessarily reflect corresponding changes at the protein or metabolic levels; thus, integrative multi‐omics strategies—including quantitative proteomics and targeted metabolomics—are essential to establish causal links between transcriptional profiles and physiological phenotypes (Storey and Storey 2012; Zhang et al. 2025). Third, functional validation of candidate genes—especially those implicated in glutathione homeostasis, autophagic flux, and sex‐dimorphic signaling pathways—is required to substantiate their regulatory roles in diapause initiation, maintenance, or termination. RNA interference (RNAi)‐mediated knockdown, coupled with phenotypic assays under controlled environmental conditions, represents a robust approach for such validation (Sim and Denlinger 2013). Fourth, and importantly, our cross‐sectional design confounds diapause duration with environmental history in two respects, neither of which can be resolved with the present data. The first is season of entry: the 0.5‐year cohort entered diapause in November (winter diapause) whereas the 1‐year and 2‐year cohorts entered in April (summer diapause). All three were induced by the same low‐temperature trigger (≤ 20°C, at which photoperiod has little influence on diapause induction in this species; Xue et al. 2002), but this establishes only that entry conditions were comparable; it does not establish that the subsequent maintenance environments were equivalent. The second, which we state plainly, is that the cohorts spent their dormancy under the ambient conditions of different calendar years—the 2‐year cohort experienced two full annual cycles, the 1‐year cohort one and the 0.5‐year cohort half of one, and the particular years differed among cohorts. Because temperature and humidity inside the outdoor containers were not continuously monitored, this exposure cannot be quantified. Duration, season of entry, number of calendar years experienced and the meteorological conditions of those years are therefore partially confounded, and the duration‐associated differences reported here should be read with that caveat rather than as a clean estimate of the effect of diapause duration alone. Any such environmental effect would, however, have to be sex‐specific in order to account for the sex‐by‐duration interaction that is the central finding of this study. A design that would break the confound is to rear cohorts under continuously monitored controlled conditions and to sample a single cohort serially at 0.5, 1 and 2 years. Fifth, survival bias is an inherent limitation: individuals that survive extended diapause (e.g., 2 years) may represent a physiologically resilient subset, potentially biasing inferences about duration‐dependent molecular reprogramming. Finally, future work must explicitly connect molecular signatures identified herein to organism‐level fitness metrics—including reproductive output, stress tolerance, and overwintering survival—to elucidate the evolutionary and ecological relevance of these regulatory strategies. Collectively, the transcriptomic resources and conceptual framework developed in this study establish a foundational platform for hypothesis‐driven research. Critically, each of the proposed hypotheses is empirically testable using well‐established methodologies—including tissue‐specific RNA interference (RNAi), integrative multi‐omics profiling, and field‐based mark–recapture experiments—thereby offering a robust pathway toward mechanistic insights into the molecular regulation of diapause.

## 5 Conclusion

This study presents the first duration‐resolved, sex‐stratified transcriptomic characterization of prolonged diapause in *Colaphellus bowringi* uncovering a dynamic molecular landscape defined by duration‐dependent transcriptional reprogramming and robust sexual dimorphism. Our findings establish diapause not as a static quiescent state, but as an actively regulated physiological condition under precise metabolic control—featuring patterns consistent with coordinated suppression of energy‐intensive processes alongside sustained transcript abundance of genes associated with cellular maintenance mechanisms essential for extended survival. Sex‐specific regulatory patterns reveal a female‐biased strategy emphasizing reproductive system stability, in contrast to a male‐biased strategy favoring transcriptional and metabolic plasticity to facilitate rapid post‐diapause reactivation. Duration profiling further resolves diapause into distinct, sequentially associated phases, suggesting a hierarchical, time‐structured regulatory program. These results refine the conceptual understanding of insect diapause by highlighting the integrated roles of environmental cues and endocrine signaling in its initiation, maintenance, and termination. To advance mechanistic insight, future studies should adopt tissue‐resolved, multi‐omics integration—including epigenomic, proteomic, and metabolomic layers—complemented by functional validation (e.g., RNAi or CRISPR‐based perturbation). Collectively, this work provides a comprehensive, sex‐ and time‐integrated mechanistic framework for prolonged diapause in *C. bowringi* and contributes broadly to the evolutionary and physiological understanding of diapause as a highly conserved, developmentally programmed life‐history adaptation in insects.

## Author Contributions

**Lili Huang:** conceptualization (equal), funding acquisition (equal), investigation (equal), writing – original draft (equal). **Yan Ni:** data curation (equal), investigation (equal). **Xin Ye:** investigation (equal). **Xiaona Liu:** investigation (equal). **Fangsen Xue:** conceptualization (equal), writing – review and editing (equal). **Haimin He:** conceptualization (equal), data curation (equal), investigation (equal).

## Funding

This project has received funding from the National Natural Science Foundation of the People's Republic of China (32360270) and High‐level Scientific Research and Innovation Team of Yuzhang Normal University (YZTD202305).

## Conflicts of Interest

The authors declare no conflicts of interest.

## Supporting information


**Figure S1:** Experimental design timeline diagram.

**Figure S2:** Hierarchical clustering heatmap of all DEGs.

**Table S1:** Summary of transcriptome assembly statistics before and after decontamination.

**Table S2:** BUSCO completeness assessment of the *de novo* transcriptome assembly.

**Table S3:** List of specific genes driving key pathways with log_2_FC and FDR values.

**Table S4:** Dispersion estimates and sequencing depth for male and female sample sets.

**Table S5:** Likelihood ratio test results for Sex × Duration interaction using the factorial DESeq2 model.

## Data Availability

The raw sequence reads have been deposited in the NCBI Sequence Read Archive under BioProject ID PRJNA1422977. The processed data supporting the main results and figures are openly available in the Dryad Digital Repository at https://doi.org/10.5061/dryad.j6q573nvn.

## References

[ece374603-bib-0001] Altschul, S. F. , W. Gish , W. Miller , E. W. Myers , and D. J. Lipman . 1990. “Basic Local Alignment Search Tool.” Journal of Molecular Biology 215: 403–410. 10.1016/S0022-2836(05)80360-2. 2231712

[ece374603-bib-0002] Buchfink, B. , C. Xie , and D. H. Huson . 2015. “Fast and Sensitive Protein Alignment Using DIAMOND.” Nature Methods 12: 59–60. 10.1038/nmeth.3176. 25402007

[ece374603-bib-0003] Camacho, C. , G. Coulouris , V. Avagyan , et al. 2009. “BLAST+: Architecture and Applications.” BMC Bioinformatics 10: 421. 10.1186/1471-2105-10-421. PMC2803857 20003500

[ece374603-bib-0004] Cedden, D. , G. Guney , S. Bayram , et al. 2025. “Multi‐Omics Analysis Reveals Diapause‐Associated Lipid Remodeling in the Fat Body of Colorado Potato Beetle.” BMC Biology 23: 294. 10.1186/s12915-025-02408-3. PMC12492533 41039468

[ece374603-bib-0005] Danks, H. V. 1987. Insect Dormancy: An Ecological Perspective. Biological Survey of Canada (Terrestrial Arthropods). https://api.semanticscholar.org/CorpusID:126844223.

[ece374603-bib-0006] Denlinger, D. L. 2002. “Regulation of Diapause.” Annual Review of Entomology 47: 93–122. 10.1146/annurev.ento.47.091201.145137. 11729070

[ece374603-bib-0007] Denlinger, D. L. 2023. “Insect Diapause: From a Rich History to an Exciting Future.” Journal of Experimental Biology 226: jeb245329. 10.1242/jeb.245329. 36852692

[ece374603-bib-0008] Dewey, C. N. , and L. Bo . 2011. “RSEM: Accurate Transcript Quantification From RNA‐Seq Data With or Without a Reference Genome.” BMC Bioinformatics 12: 323. 10.1186/1471-2105-12-323. PMC3163565 21816040

[ece374603-bib-0009] Easwaran, S. , and D. J. Montell . 2023. “The Molecular Mechanisms of Diapause and Diapause‐Like Reversible Arrest.” Biochemical Society Transactions 51: 1847–1856. 10.1042/BST20221431. PMC10657177 37800560

[ece374603-bib-0010] Eddy, S. R. 1998. “Profile Hidden Markov Models.” Bioinformatics 14: 755–763. 10.1093/bioinformatics/14.9.755. 9918945

[ece374603-bib-0011] Elbein, A. D. , Y. T. Pan , I. Pastuszak , and D. Carroll . 2003. “New Insights on Trehalose: A Multifunctional Molecule.” Glycobiology 13: 17R–27R. 10.1093/glycob/cwg047. 12626396

[ece374603-bib-0012] Flatt, T. , and A. Heyland . 2011. Mechanisms of Life History Evolution: The Genetics and Physiology of Life History Traits and Trade‐Offs. Oxford University Press. 10.1093/acprof:oso/9780199568765.001.0001.

[ece374603-bib-0013] Grabherr, M. G. , B. J. Haas , M. Yassour , et al. 2011. “Full‐Length Transcriptome Assembly From RNA‐Seq Data Without a Reference Genome.” Nature Biotechnology 29: 644–652. 10.1038/nbt.1883. PMC3571712 21572440

[ece374603-bib-0014] Güney, G. , D. Cedden , J. Körnig , et al. 2024. “Physiological and Transcriptional Changes Associated With Obligate Aestivation in the Cabbage Stem Flea Beetle (*Psylliodes chrysocephala*).” Insect Biochemistry and Molecular Biology 173: 104165. 10.1016/j.ibmb.2024.104165. 39089389

[ece374603-bib-0015] Güney, G. , D. Cedden , S. Scholten , and M. Rostás . 2025. “Reciprocal Roles of Two Trehalose Transporters in Aestivating Cabbage Stem Flea Beetle (*Psylliodes chrysocephala*).” Insect Biochemistry and Molecular Biology 180: 104308. 10.1016/j.ibmb.2025.104308. 40174677

[ece374603-bib-0016] Güney, G. , D. Cedden , S. Scholten , and M. Rostás . 2026. “Proteomic Changes Associated With the Initiation and Termination of Aestivation in the Cabbage Stem Flea Beetle.” Journal of Insect Physiology 169: 104955. 10.1016/j.jinsphys.2026.104955. 41724350

[ece374603-bib-0017] Hahn, D. A. , and D. L. Denlinger . 2011. “Energetics of Insect Diapause.” Annual Review of Entomology 56: 103–121. 10.1146/annurev-ento-112408-085436. 20690828

[ece374603-bib-0018] Hand, S. C. , D. L. Denlinger , J. E. Podrabsky , and R. Roy . 2016. “Mechanisms of Animal Diapause: Recent Developments From Nematodes, Crustaceans, Insects, and Fish.” American Journal of Physiology. Regulatory, Integrative and Comparative Physiology 310: R1193–R1211. 10.1152/ajpregu.00250.2015. PMC4935499 27053646

[ece374603-bib-0019] Hanski, I. 1988. “Four Kinds of Extra Long Diapause in Insects: A Review of Theory and Observations.” Annales Zoologici Fennici 25: 37–53. https://www.jstor.org/stable/23734707.

[ece374603-bib-0020] Izadi, H. 2025. “Endocrine and Enzymatic Shifts During Insect Diapause: A Review of Regulatory Mechanisms.” Frontiers in Physiology 16: 1544198. 10.3389/fphys.2025.1544198. PMC11949959 40161974

[ece374603-bib-0021] Koštál, V. 2006. “Eco‐Physiological Phases of Insect Diapause.” Journal of Insect Physiology 52: 113–127. 10.1016/j.jinsphys.2005.09.008. 16332347

[ece374603-bib-0022] Langmead, B. , C. Trapnell , M. Pop , and S. L. Salzberg . 2009. “Ultrafast and Memory‐Efficient Alignment of Short DNA Sequences to the Human Genome.” Genome Biology 10: R25. 10.1186/gb-2009-10-3-r25. PMC2690996 19261174

[ece374603-bib-0023] Lecheta, M. C. , C. Nielson , B. W. French , E. A. W. Nadeau , and N. M. Teets . 2025. “Molecular Mechanisms and Comparative Transcriptomics of Diapause in Two Corn Rootworm Species (*Diabrotica* spp.).” Current Research in Insect Science 7: 100104. 10.1016/j.cris.2024.100104. PMC11786089 39895870

[ece374603-bib-0024] Li, J. X. , Z. Cao , S. Guo , et al. 2020. “Molecular Characterization and Functional Analysis of Two Trehalose Transporter Genes in the Cabbage Beetle, *Colaphellus bowringi* .” Journal of Asia‐Pacific Entomology 23: 627–633. 10.1016/j.aspen.2020.05.011.

[ece374603-bib-0025] Liu, Z. D. , Y. C. Xin , Y. N. Zhang , J. T. Fan , and J. H. Sun . 2016. “Summer Diapause Induced by High Temperatures in the Oriental Tobacco Budworm: Ecological Adaptation to Hot Summers.” Scientific Reports 6: 27443. 10.1038/srep27443. PMC4895216 27271223

[ece374603-bib-0026] Love, M. I. , W. Huber , and S. Anders . 2014. “Moderated Estimation of Fold Change and Dispersion for RNA‐Seq Data With DESeq2.” Genome Biology 15: 550. 10.1186/s13059-014-0550-8. PMC4302049 25516281

[ece374603-bib-0027] MacRae, T. H. 2010. “Gene Expression, Metabolic Regulation and Stress Tolerance During Diapause.” Cellular and Molecular Life Sciences 67: 2405–2424. 10.1007/s00018-010-0311-0. PMC11115916 20213274

[ece374603-bib-0028] Margus, A. , and L. Lindström . 2020. “Prolonged Diapause Has Sex‐Specific Fertility and Fitness Costs.” Evolutionary Ecology 34: 41–57. 10.1007/s10682-019-10024-1.

[ece374603-bib-0029] Matsuo, Y. 2006. “Cost of Prolonged Diapause and Its Relationship to Body Size in a Seed Predator.” Functional Ecology 20: 300–306. 10.1111/j.1365-2435.2006.01097.x.

[ece374603-bib-0030] Menu, F. 1993. “Strategies of Emergence in the Chestnut Weevil *Curculio elephas* (Coleoptera: Curculionidae).” Oecologia 96: 383–390. 10.1007/BF00317509. 28313654

[ece374603-bib-0031] Ni, Y. , Y. F. Li , F. S. Xue , L. L. Huang , and H. M. He . 2025. “Effect of Diapause Duration on Thermal Tolerance in the Cabbage Beetle *Colaphellus bowringi* Baly.” Journal of Insect Physiology 167: 104912. 10.1016/j.jinsphys.2025.104912. 41274544

[ece374603-bib-0032] Poelchau, M. F. , J. A. Reynolds , C. G. Elsik , D. L. Denlinger , and P. A. Armbruster . 2013. “RNA‐Seq Reveals Early Distinctions and Late Convergence of Gene Expression Between Diapause and Quiescence in the Asian Tiger Mosquito, *Aedes albopictus* .” Journal of Experimental Biology 216: 4082–4090. 10.1242/jeb.089508. PMC3797908 23913949

[ece374603-bib-0033] Ragland, G. J. , P. A. Armbruster , and M. E. Meuti . 2019. “Evolutionary and Functional Genetics of Insect Diapause: A Call for Greater Integration.” Current Opinion in Insect Science 36: 74–81. 10.1016/j.cois.2019.08.003. PMC7212789 31539788

[ece374603-bib-0034] Roncalli, V. , S. A. Sommer , M. C. Cieslak , C. Cheryl , R. R. Hopcroft , and P. H. Lenz . 2018. “Physiological Characterization of the Emergence From Diapause: A Transcriptomics Approach.” Scientific Reports 8: 12577. 10.1038/s41598-018-30873-0. PMC6105596 30135598

[ece374603-bib-0035] Sim, C. , and D. L. Denlinger . 2013. “Insulin Signaling and the Regulation of Insect Diapause.” Frontiers in Physiology 4: 189. 10.3389/fphys.2013.00189. PMC3717507 23885240

[ece374603-bib-0036] Sinclair, B. J. , and K. E. Marshall . 2018. “The Many Roles of Fats in Overwintering Insects.” Journal of Experimental Biology 221: jeb161836. 10.1242/jeb.161836. 29514877

[ece374603-bib-0037] Šlachta, M. , J. Vambera , and H. Zahradníčková . 2002. “Entering Diapause Is a Prerequisite for Successful Cold‐Acclimation in Adult *Graphosoma lineatum* (Heteroptera: Pentatomidae).” Journal of Insect Physiology 48: 1031–1039. 10.1016/S0022-1910(02)00191-9. 12770026

[ece374603-bib-0038] Soula, B. , and F. Menu . 2003. “Variability in Diapause Duration in the Chestnut Weevil: Mixed ESS, Genetic Polymorphism or Bet‐Hedging?” Oikos 100: 574–580. 10.1034/j.1600-0706.2003.11858.x.

[ece374603-bib-0039] Storey, K. B. , and J. M. Storey . 2012. “Insect Cold Hardiness: Metabolic, Gene, and Protein Adaptation.” Canadian Journal of Zoology 90: 456–475. 10.1139/z2012-011.

[ece374603-bib-0040] Tang, J. J. , H. M. He , C. Chen , S. Fu , and F. S. Xue . 2017. “Latitudinal Cogradient Variation of Development Time and Growth Rate and a Negative Latitudinal Body Weight Cline in a Widely Distributed Cabbage Beetle.” PLoS One 12: e0181030. 10.1371/journal.pone.0181030. PMC5507546 28704496

[ece374603-bib-0041] Tang, J. J. , X. P. Liu , H. M. He , L. L. Huang , S. H. Wu , and F. S. Xue . 2022. “A Multi‐Year Dormancy Strategy in a Cabbage Beetle Population in Southeastern China.” Ecology and Evolution 12: e8900. 10.1002/ece3.8900. PMC9077826 35571750

[ece374603-bib-0042] Tauber, M. J. , and C. A. Tauber . 2002. “Prolonged Dormancy in *Leptinotarsa decemlineata* (Say) (Coleoptera: Chrysomelidae): A Ten‐Year Field Study With Implications for Crop Rotation.” Environmental Entomology 31: 499–504. 10.1603/0046-225X-31.3.499.

[ece374603-bib-0043] Tian, Z. , K. Wang , S. Guo , et al. 2025. “The PBAP Chromatin Remodeling Complex Mediates Summer Diapause via H3K4me3‐Driven Juvenile Hormone Regulation in *Colaphellus bowringi* .” Proceedings of the National Academy of Sciences of the United States of America 122: e2422328122. 10.1073/pnas.2422328122. PMC11962415 40112108

[ece374603-bib-0044] Torson, A. S. , D. Melicher , G. D. Yocum , and J. P. Rinehart . 2026. “Diapause in the Colorado Potato Beetle Is Characterized by Sex‐Specific Gene Expression.” Journal of Insect Physiology 171: 104990. 10.1016/j.jinsphys.2026.104990. 41999911

[ece374603-bib-0045] Wang, X. P. , F. S. Xue , A. Hua , and F. Ge . 2006. “Effects of Diapause Duration on Future Reproduction in the Cabbage Beetle, *Colaphellus bowringi*: Positive or Negative?” Physiological Entomology 31: 190–196. 10.1111/j.1365-3032.2006.00508.x.

[ece374603-bib-0046] Xiao, Y. , Q. Li , Z. Sun , et al. 2025. “Transcriptomics and Metabolomics Combined to Analyze the Response Mechanism of Silkworm Eggs to High‐Temperature Stress.” Insects 16: 862. 10.3390/insects16080862. PMC12386467 40870663

[ece374603-bib-0047] Xu, R. , Y. H. Chen , J. F. Xia , T. X. Zeng , Y. H. Li , and J. Y. Zhu . 2021. “Metabolic Dynamics Across Prolonged Diapause Development in Larvae of the Sawfly, *Cephalcia chuxiongica* (Hymenoptera: Pamphiliidae).” Journal of Asia‐Pacific Entomology 24: 1–6. 10.1016/j.aspen.2021.04.015.

[ece374603-bib-0048] Xue, F. S. , H. R. Spieth , A. Q. Li , and A. Hua . 2002. “The Role of Photoperiod and Temperature in Determination of Summer and Winter Diapause in the Cabbage Beetle, *Colaphellus bowringi* (Coleoptera: Chrysomelidae).” Journal of Insect Physiology 48: 279–286. 10.1016/S0022-1910(01)00172-x. 12770101

[ece374603-bib-0049] Yu, G. , L. G. Wang , Y. Han , and Q. Y. He . 2012. “clusterProfiler: An R Package for Comparing Biological Themes Among Gene Clusters.” OMICS: A Journal of Integrative Biology 16: 284–287. 10.1089/omi.2011.0118. PMC3339379 22455463

[ece374603-bib-0050] Zhang, M. S. , W. W. He , Y. Y. Li , J. J. Chen , N. M. Teets , and L. S. Zhang . 2025. “Metabolic and Transcriptional Regulation of Reproductive Diapause in *Arma chinensis* .” iScience 28: 111761. 10.1016/j.isci.2025.111761. PMC11928864 40124477

[ece374603-bib-0051] Zhu, L. , Z. Tian , S. Guo , W. Liu , F. Zhu , and X. P. Wang . 2019. “Circadian Clock Genes Link Photoperiodic Signals to Lipid Accumulation During Diapause Preparation in the Diapause‐Destined Female Cabbage Beetles *Colaphellus bowringi* .” Insect Biochemistry and Molecular Biology 104: 1–10. 10.1016/j.ibmb.2018.11.001. 30423421

