# Supplementary material for: Molecular Adaptations and Sex‐Biased Regulatory Pathways in Multi‐Year Diapause of the Cabbage Beetle Colaphellus bowringi: A Duration‐Resolved Transcriptomic Analysis
Source: Ecol Evol. 2026 Oct 28;16(11):e74603. doi: 10.1002/ece3.74603 (PMC13714628; doi:10.1002/ece3.74603)
Supplement: Supplementary file 1 [file ECE3-16-e74603-s001.docx]

Table S2. BUSCO completeness assessment of the Colaphellus bowringi transcriptome assembly

| BUSCO metric | Count | Percentage (%) |
| --- | --- | --- |
| Complete BUSCOs (C) | 1,297 | 94.9 |
| Complete and single-copy (S) | 1,289 | 94.3 |
| Complete and duplicated (D) | 8 | 0.6 |
| Fragmented BUSCOs (F) | 24 | 1.8 |
| Missing BUSCOs (M) | 46 | 3.3 |
| Total BUSCO groups searched | 1,367 | 100 |

Note: BUSCO v5.2.1 was run in genome mode against the insecta_odb10 lineage dataset (creation date 2019-11-20; 75 genomes; 1,367 BUSCO groups), with AUGUSTUS v3.1.0 as the gene predictor. C, complete; S, complete and single-copy; D, complete and duplicated; F, fragmented; M, missing.
