# Supplementary material for: Molecular Adaptations and Sex‐Biased Regulatory Pathways in Multi‐Year Diapause of the Cabbage Beetle Colaphellus bowringi: A Duration‐Resolved Transcriptomic Analysis
Source: Ecol Evol. 2026 Oct 28;16(11):e74603. doi: 10.1002/ece3.74603 (PMC13714628; doi:10.1002/ece3.74603)
Supplement: Supplementary file 2 [file ECE3-16-e74603-s002.docx]

**Table S4. Comparison of DESeq2 dispersion estimates and sequencing depth between male and female sample groups**

| **Sex** | **Sample Group** | **Median Dispersion** | **IQR** | **Mean Mapped Reads (M)** | **Range (M)** |
| --- | --- | --- | --- | --- | --- |
| Female | F0.5 | — | — | 20.78 | 19.10 – 22.04 |
| Female | F1.0 | — | — | 20.21 | 18.37 – 21.72 |
| Female | F2.0 | — | — | 18.68 | 17.87 – 19.71 |
| Male | M0.5 | — | — | 21.44 | 18.78 – 23.03 |
| Male | M1.0 | — | — | 19.28 | 16.55 – 21.78 |
| Male | M2.0 | — | — | 19.67 | 18.06 – 20.73 |
| Overall | Females | 2.826 | 9.799 | 19.89 | 17.87 – 22.04 |
|  | Males | 8.755 | 9.598 | 20.13 | 16.55 – 23.03 |

Note: Dispersion estimates are gene-level medians computed across all genes within each sex. “—” indicates that dispersion was not separately calculated for each duration group due to limited sample size (n=6 per group); the combined sex-level estimates provide the most robust comparison between males and females.
