# Supplementary material for: Molecular Adaptations and Sex‐Biased Regulatory Pathways in Multi‐Year Diapause of the Cabbage Beetle Colaphellus bowringi: A Duration‐Resolved Transcriptomic Analysis
Source: Ecol Evol. 2026 Oct 28;16(11):e74603. doi: 10.1002/ece3.74603 (PMC13714628; doi:10.1002/ece3.74603)
Supplement: Supplementary file 3 [file ECE3-16-e74603-s003.docx]

## Table S1. Assembly statistics of the Colaphellus bowringi transcriptome before and after decontamination

| Metric | Initial assembly | After decontamination |
| --- | --- | --- |
| Total number of Unigenes | 66,379 | 50,247 |
| Total length (bp) | 55,438,785 | 45,728,806 |
| N50 length (bp) | 1,598 | 1,886 |
| Mean length (bp) | 835.19 | 910.08 |
| Number of Unigenes ≥ 1 kb | 14,680 (22.12%) | 12,509 (24.90%) |
| GC content (%) | — | 37.79 |
| Unigenes removed as non-arthropod contamination | — | 16,132 (24.3%) |
| of which Bacteria | — | 16,097 |
| of which Fungi | — | 35 |
| Mean length of removed Unigenes (bp) | — | 601.91 |
| N50 of removed Unigenes (bp) | — | 759 |
| GC content of removed Unigenes (%) | — | 55.18 |

Note: The initial Trinity assembly comprised 66,379 Unigenes. Decontamination was performed by BLASTn alignment of all Unigenes against the NCBI NT database (E-value ≤ 1e-5); Unigenes whose best hit was a non-arthropod organism (Bacteria or Fungi) were removed. This removed 16,132 Unigenes (24.3% of the initial assembly; 16,097 Bacteria and 35 Fungi), leaving 50,247 clean Unigenes that were used for all downstream annotation, quantification and differential expression analyses. The removed fraction was markedly shorter and had a much higher GC content than the retained set, a compositional signature characteristic of bacterial sequences.
