# Supplementary material for: Molecular Adaptations and Sex‐Biased Regulatory Pathways in Multi‐Year Diapause of the Cabbage Beetle Colaphellus bowringi: A Duration‐Resolved Transcriptomic Analysis
Source: Ecol Evol. 2026 Oct 28;16(11):e74603. doi: 10.1002/ece3.74603 (PMC13714628; doi:10.1002/ece3.74603)
Supplement: Supplementary file 4 [file ECE3-16-e74603-s004.docx]

**Table S3. List of representative differentially expressed genes (DEGs) driving key enriched KEGG pathways and GO terms**

| **Pathway / GO Term** | **Unigene ID** | **Gene Annotation (NR Hit)** | **Sex / Comparison** | **log₂ (Fold Change)** | **FDR-adjusted p-value** | **Transcript Abundance Direction** |
| --- | --- | --- | --- | --- | --- | --- |
| Glutathione metabolism (ko00480) | TRINITY_DN13374_c0_g1 | Transposon Ty3-I Gag-Pol polyprotein [Labeo rohita] (KO K00432, glutathione peroxidase) | F0.5 vs F1.0 | +2.605 | 2.07×10⁻⁵ | Increased |
| Glutathione metabolism (ko00480) | TRINITY_DN4850_c1_g1 | Retrovirus-related Pol polyprotein from transposon opus [Triplophysa tibetana] (KO K00432) | F0.5 vs F1.0 | −1.855 | 2.56×10⁻³ | Decreased |
| Glutathione metabolism (ko00480) | TRINITY_DN6833_c0_g1 | Glutathione hydrolase 1 proenzyme [Petromyzon marinus] (KO K18592) | F0.5 vs F1.0 | +3.606 | 1.31×10⁻³ | Increased |
| Lysosome (ko04142) | TRINITY_DN20916_c0_g1 | Cathepsin L1-like [Zootoca vivipara] | F0.5 vs F1.0 | +1.682 | 6.23×10⁻⁶ | Increased |
| Lysosome (ko04142) | TRINITY_DN6390_c0_g1 | Cathepsin B [Scophthalmus maximus] | F0.5 vs F1.0 | +1.497 | 8.91×10⁻³ | Increased |
| Lysosome (ko04142) | TRINITY_DN2678_c0_g1 | Lysosomal acid lipase/cholesteryl ester hydrolase-like [Acipenser ruthenus] | F0.5 vs F1.0 | +1.579 | 3.06×10⁻⁵ | Increased |
| Lysosome (ko04142) | TRINITY_DN705_c0_g1 | Sialin-like [Petromyzon marinus] (SLC17A5) | F0.5 vs F1.0 | −1.768 | 1.98×10⁻⁶ | Decreased |
| Autophagy – animal (ko04140) | TRINITY_DN2266_c0_g1 | Cathepsin D [Rachycentron canadum] | F0.5 vs F1.0 | +1.588 | 1.25×10⁻⁶ | Increased |
| Autophagy – animal (ko04140) | TRINITY_DN6390_c0_g1 | Cathepsin B [Scophthalmus maximus] | F0.5 vs F1.0 | +1.497 | 8.91×10⁻³ | Increased |
| Wnt signaling pathway (ko04310) | TRINITY_DN9522_c0_g2 | Core-binding factor subunit beta isoform X2 [Xenopus tropicalis] | F0.5 vs F1.0 | +1.480 | 9.74×10⁻³ | Increased |
| Toll and Imd signaling pathway (ko04624) | TRINITY_DN7001_c0_g1 | Peptidoglycan-recognition protein SC2-like [Trachemys scripta elegans] | F0.5 vs F1.0 | +1.283 | 1.30×10⁻³ | Increased |
| Toll and Imd signaling pathway (ko04624) | TRINITY_DN9112_c0_g1 | Peptidoglycan-recognition protein SC2-like [Chrysemys picta bellii] | F0.5 vs F1.0 | +2.957 | 8.96×10⁻²² | Increased |
| Reproductive process (GO:0000003) | TRINITY_DN2869_c0_g1 | Vitellogenin-1-like [Buceros rhinoceros silvestris] | F0.5 vs F1.0 | −1.599 | 2.37×10⁻³ | Decreased |
| Reproductive process (GO:0000003) | TRINITY_DN13443_c0_g1 | Alpha-tocopherol transfer protein [Podarcis muralis] | F0.5 vs F1.0 | −1.937 | 3.48×10⁻⁸ | Decreased |
| Glutathione metabolism (ko00480) | TRINITY_DN126_c0_g1 | Glutathione S-transferase 1-like [Petromyzon marinus] (K00799) | F0.5 vs F2.0 | +1.031 | 3.46×10⁻⁴ | Increased |
| Glutathione metabolism (ko00480) | TRINITY_DN2991_c0_g1 | Uncharacterized protein K02A2.6-like [Oreochromis niloticus] (K00432, glutathione peroxidase) | F0.5 vs F2.0 | +1.478 | 9.26×10⁻⁴ | Increased |
| Lysosome (ko04142) | TRINITY_DN14403_c0_g1 | CD63 antigen [Maylandia zebra] (K06497) | F0.5 vs F2.0 | −9.374 | 2.29×10⁻¹¹ | Decreased |
| Lysosome (ko04142) | TRINITY_DN3954_c0_g1 | Sialin [Petromyzon marinus] (SLC17A5, K12301) | F0.5 vs F2.0 | +1.049 | 1.35×10⁻³ | Increased |
| Lysosome (ko04142) | TRINITY_DN4709_c0_g1 | Cathepsin B [Scophthalmus maximus] (K01363) | F0.5 vs F2.0 | +1.241 | 5.89×10⁻⁵ | Increased |
| Autophagy – animal (ko04140) | TRINITY_DN14612_c0_g1 | Cathepsin L1-like [Takifugu rubripes] (K01365) | F0.5 vs F2.0 | −13.79 | 2.82×10⁻⁹ | Decreased |
| Toll-like receptor signaling pathway (ko04620) | TRINITY_DN50791_c0_g1 | Toll-like receptor 1 [Pelodiscus sinensis] (K05398) | F0.5 vs F2.0 | +4.339 | 6.76×10⁻⁵ | Increased |
| Reproductive process (GO:0000003) | TRINITY_DN896_c0_g1 | Farnesyl pyrophosphate synthase [Oryzias latipes] (K00787) | F0.5 vs F2.0 | +1.436 | 7.05×10⁻¹⁵ | Increased |
| Glutathione metabolism (ko00480) | TRINITY_DN2991_c0_g1 | Uncharacterized protein K02A2.6-like [Oreochromis niloticus] (K00432) | M0.5 vs M1.0 | +1.532 | 9.50×10⁻⁶ | Increased |
| Glutathione metabolism (ko00480) | TRINITY_DN12400_c0_g1 | Retrovirus-related Pol polyprotein from transposon opus [Triplophysa tibetana] (K00432) | M0.5 vs M1.0 | −2.662 | 2.33×10⁻³ | Decreased |
| Lysosome (ko04142) | TRINITY_DN20010_c0_g1 | Lysosomal acid phosphatase [Salmo trutta] (K14410) | M0.5 vs M1.0 | +2.369 | 1.84×10⁻⁸ | Increased |
| Lysosome (ko04142) | TRINITY_DN6310_c0_g1 | Cathepsin L1-like [Colinus virginianus] (K01365) | M0.5 vs M1.0 | −1.143 | 1.42×10⁻¹¹ | Decreased |
| Autophagy – animal (ko04140) | TRINITY_DN6310_c0_g1 | Cathepsin L1-like [Colinus virginianus] (K01365) | M0.5 vs M1.0 | −1.143 | 1.42×10⁻¹¹ | Decreased |
| ECM-receptor interaction (ko04512) | TRINITY_DN5204_c0_g1 | Laminin subunit alpha-5 isoform X3 [Terrapene carolina triunguis] (K06240) | M0.5 vs M1.0 | +1.180 | 8.56×10⁻⁸ | Increased |
| ABC transporters (ko02010) | TRINITY_DN7396_c0_g1 | ATP-binding cassette sub-family G member 4-like [Callorhinchus milii] (K05679) | M0.5 vs M1.0 | −1.463 | 3.32×10⁻¹⁰ | Decreased |
| Retinol metabolism (ko00830) | TRINITY_DN6993_c0_g1 | Cytochrome P450 27C1-like [Erpetoichthys calabaricus] (K17951) | M0.5 vs M1.0 | +3.331 | 8.25×10⁻⁴ | Increased |
| Retinol metabolism (ko00830) | TRINITY_DN17576_c0_g1 | Retinol dehydrogenase 11-like [Oncorhynchus nerka] (K11153) | M0.5 vs M1.0 | −1.270 | 1.21×10⁻³ | Decreased |
| Glycerolipid metabolism (ko00561) | TRINITY_DN10453_c0_g1 | Pancreatic triacylglycerol lipase-like [Callorhinchus milii] (K14073) | M0.5 vs M1.0 | +4.616 | 7.12×10⁻¹⁶ | Increased |
| Glycerolipid metabolism (ko00561) | TRINITY_DN340_c1_g1 | Lipase member I [Geotrypetes seraphini] (K14074) | M0.5 vs M1.0 | −1.362 | 1.75×10⁻⁵ | Decreased |
| Protein processing in ER (ko04141) | TRINITY_DN20216_c0_g1 | 78 kDa glucose-regulated protein [Paramormyrops kingsleyae] (K09490) | M0.5 vs M1.0 | +1.287 | 1.55×10⁻⁸ | Increased |
| Protein processing in ER (ko04141) | TRINITY_DN8192_c0_g1 | Endoplasmin [Pogona vitticeps] (K09487) | M0.5 vs M1.0 | +1.044 | 1.63×10⁻⁷ | Increased |
| Reproductive process (GO:0000003) | TRINITY_DN3098_c0_g1 | Arf-GAP domain and FG repeat-containing protein 1-like [Fundulus heteroclitus] (K15044) | M0.5 vs M1.0 | +1.088 | 7.79×10⁻⁶ | Increased |
| Glutathione metabolism (ko00480) | TRINITY_DN6164_c1_g1 | Glutathione peroxidase [Triplophysa tibetana] (K00432) | M0.5 vs M2.0 | +4.615 | 1.02×10⁻⁵ | Increased |
| Glutathione metabolism (ko00480) | TRINITY_DN6438_c0_g1 | Glutathione peroxidase [Oreochromis niloticus] (K00432) | M0.5 vs M2.0 | −1.694 | 6.31×10⁻³ | Decreased |
| Glutathione metabolism (ko00480) | TRINITY_DN12400_c0_g1 | Glutathione peroxidase [Triplophysa tibetana] (K00432) | M0.5 vs M2.0 | −3.896 | 9.90×10⁻⁷ | Decreased |
| Lysosome (ko04142) | TRINITY_DN20010_c0_g1 | Lysosomal acid phosphatase [Salmo trutta] (K14410) | M0.5 vs M2.0 | +1.648 | 4.66×10⁻³ | Increased |
| Lysosome (ko04142) | TRINITY_DN11333_c0_g1 | Cathepsin L [Scleropages formosus] (K01365) | M0.5 vs M2.0 | +9.657 | 1.82×10⁻¹⁰ | Increased |
| Lysosome (ko04142) | TRINITY_DN20720_c0_g1 | Cathepsin L [Takifugu bimaculatus] (K01365) | M0.5 vs M2.0 | +1.133 | 9.77×10⁻⁵ | Increased |
| Lysosome (ko04142) | TRINITY_DN526_c0_g1 | Prostatic acid phosphatase [Xenopus laevis] (K19283) | M0.5 vs M2.0 | −1.413 | 1.23×10⁻⁷ | Decreased |
| Autophagy – animal (ko04140) | TRINITY_DN11333_c0_g1 | Cathepsin L [Scleropages formosus] (K01365) | M0.5 vs M2.0 | +9.657 | 1.82×10⁻¹⁰ | Increased |
| Autophagy – animal (ko04140) | TRINITY_DN20720_c0_g1 | Cathepsin L [Takifugu bimaculatus] (K01365) | M0.5 vs M2.0 | +1.133 | 9.77×10⁻⁵ | Increased |
| ECM-receptor interaction (ko04512) | TRINITY_DN6436_c0_g1 | Low-density lipoprotein receptor-related protein 4-like [Petromyzon marinus] (K04550) | M0.5 vs M2.0 | +4.945 | 4.03×10⁻³ | Increased |
| ECM-receptor interaction (ko04512) | TRINITY_DN4503_c0_g1 | Cubilin [Lepisosteus oculatus] (K14616) | M0.5 vs M2.0 | −1.429 | 2.10×10⁻³ | Decreased |
| ABC transporters (ko02010) | TRINITY_DN6341_c0_g1 | Canalicular multispecific organic anion transporter 2 [Nothoprocta perdicaria] (K05665) | M0.5 vs M2.0 | +1.810 | 4.13×10⁻³ | Increased |
| ABC transporters (ko02010) | TRINITY_DN2272_c0_g1 | Multidrug resistance-associated protein 4 [Carassius auratus] (K05673) | M0.5 vs M2.0 | −2.156 | 7.26×10⁻⁵ | Decreased |
| ABC transporters (ko02010) | TRINITY_DN8536_c0_g1 | ATP-binding cassette sub-family G member 2 [Parambassis ranga] (K21396) | M0.5 vs M2.0 | −1.051 | 5.92×10⁻³ | Decreased |
| Glycerolipid metabolism (ko00561) | TRINITY_DN10453_c0_g1 | Pancreatic triacylglycerol lipase [Callorhinchus milii] (K14073) | M0.5 vs M2.0 | +4.359 | 2.22×10⁻⁴ | Increased |
| Glycerolipid metabolism (ko00561) | TRINITY_DN953_c0_g1 | Pancreatic lipase-related protein 1 [Dasyatis pastinaca] (K14073) | M0.5 vs M2.0 | +1.092 | 1.77×10⁻⁶ | Increased |
| Protein processing in ER (ko04141) | TRINITY_DN6167_c0_g1 | Alpha-crystallin B chain [Carassius auratus] (K09542) | M0.5 vs M2.0 | +1.922 | 9.42×10⁻⁵ | Increased |
| Protein processing in ER (ko04141) | TRINITY_DN7821_c0_g1 | Alpha-crystallin B chain [Chanos chanos] (K09542) | M0.5 vs M2.0 | +1.236 | 3.68×10⁻³ | Increased |
| Retinol metabolism (ko00830) | TRINITY_DN14518_c1_g1 | Cytochrome P450 4V2 [Serinus canaria] (K15001) | M0.5 vs M2.0 | −3.271 | 1.39×10⁻⁴ | Decreased |
| Retinol metabolism (ko00830) | TRINITY_DN3848_c0_g1 | Cytochrome P450 4V2 [Erpetoichthys calabaricus] (K15001) | M0.5 vs M2.0 | −2.092 | 1.02×10⁻⁹ | Decreased |
| Reproductive process (GO:0000003) | TRINITY_DN2869_c0_g1 | Vitellogenin-1-like [Buceros rhinoceros silvestris] | M0.5 vs M2.0 | +7.394 | 5.21×10⁻⁴ | Increased |
| Reproductive process (GO:0000003) | TRINITY_DN528_c0_g1 | Vitellogenin-1-like [Merops nubicus] | M0.5 vs M2.0 | +7.685 | 4.32×10⁻⁴ | Increased |
| Reproductive process (GO:0000003) | TRINITY_DN812_c0_g1 | Alpha-tocopherol transfer protein [Geotrypetes seraphini] (K19625) | M0.5 vs M2.0 | +1.508 | 1.92×10⁻³ | Increased |
