# Supplementary material for: Molecular Adaptations and Sex‐Biased Regulatory Pathways in Multi‐Year Diapause of the Cabbage Beetle Colaphellus bowringi: A Duration‐Resolved Transcriptomic Analysis
Source: Ecol Evol. 2026 Oct 28;16(11):e74603. doi: 10.1002/ece3.74603 (PMC13714628; doi:10.1002/ece3.74603)
Supplement: Supplementary file 5 [file ECE3-16-e74603-s005.docx]

Table S5. Likelihood ratio test (LRT) for the significance of the Sex x Duration interaction on gene expression

| Parameter | Value |
| --- | --- |
| Full model | ~ Sex + Duration + Sex:Duration |
| Reduced model | ~ Sex + Duration |
| Test statistic | Likelihood ratio statistic, 2 x (logLik_full - logLik_reduced) |
| Null distribution | Chi-square, 1 degree of freedom |
| Number of genes tested | 50,247 |
| Significant interaction genes (FDR < 0.05) | 2,352 |
| LRT statistic (range) | 0.00 - 157.60 |
| Median LRT statistic (all genes) | 3.41 |
| Raw p-value (range) | 1.30 x 10^-36 - 1.00 |
| Variance stabilising transformation | vst, blind = FALSE |
| Multiple-testing correction | Benjamini-Hochberg |
| Interpretation | The Sex x Duration interaction significantly improves model fit for 2,352 genes, supporting the conclusion that the transcriptional response to diapause duration differs between males and females. |

Note: The LRT compared a full DESeq2 model (~ Sex + Duration + Sex:Duration) against a reduced model without the interaction term (~ Sex + Duration). A likelihood ratio statistic is defined as 2 x (log-likelihood of the full model - log-likelihood of the reduced model) and is therefore non-negative by construction; any negative value indicates a numerical/convergence artefact rather than a genuine test result. Where the optimiser returned a transiently negative statistic (reduced model fitting marginally better), the value was truncated to zero before computing the p-value from the chi-square distribution with 1 degree of freedom. Genes for which the full model did not converge were excluded (n = 0 excluded). The interaction was considered significant at an FDR-adjusted p < 0.05.
